# Synthesis of novel coumarin-based thiosemicarbazones and their implications in diabetic management via in-vitro and in-silico approaches

**DOI:** 10.1038/s41598-023-44837-6

**Published:** 2023-10-21

**Authors:** Syeda Bakhtawar Zahra, Saeed Ullah, Sobia Ahsan Halim, Muhammad Waqas, Noor Ul Huda, Ajmal Khan, Ammena Y. Binsaleh, Attalla F. El-kott, Javid Hussain, Ahmed Al-Harrasi, Zahid Shafiq

**Affiliations:** 1 Institute of Chemical Sciences, Bahauddin Zakariya University, Multan, 60800 Pakistan; 2 Natural and Medical Sciences Research Centre, University of Nizwa, Birkat Al Mauz, P.O. Box 33, 616 Nizwa, Oman; 3 Department of Pharmacy Practice, College of Pharmacy, Princess Nourah Bint Abdulrahman University, P.O. Box 84428, 11671 Riyadh, Saudi Arabia; 4 Department of Biology, College of Science, King Khalid University, 61421 Abha, Saudi Arabia; 5 Department of Zoology, College of Science, Damanhour University, Damanhour, 22511 Egypt; 6 Department of Biological Sciences & Chemistry, College of Arts and Sciences, University of Nizwa, Nizwa, 616 Oman

**Keywords:** Medicinal chemistry, Organic chemistry, NMR spectroscopy

## Abstract

Diabetes mellitus has a high prevalence rate and it has been deemed a severe chronic metabolic disorder with long-term complications. This research aimed to identify compounds that could potentially inhibit the vital metabolic enzyme α-glucosidase and thereby exert an anti-hyperglycemic effect. The main goal was to establish an effective approach to control diabetes. To proceed with this study, a series of novel coumarin-derived thiosemicarbazones **3a**–**3m** was synthesized and examined using a variety of spectroscopic methods. Moreover, all the compounds were subjected to α-glucosidase inhibition bioassay to evaluate their antidiabetic potential. Fortunately, all the compounds exhibited several folds potent α-glucosidase inhibitory activities with IC_50_ values ranging from 2.33 to 22.11 µM, in comparison to the standard drug acarbose (IC_50_ = 873.34 ± 1.67 µM). The kinetic studies of compound **3c** displayed concentration-dependent inhibition. Furthermore, the binding modes of these molecules were elucidated through a molecular docking strategy which depicted that the thiosemicarbazide moiety of these molecules plays a significant role in the interaction with different residues of the α-glucosidase enzyme. However, their conformational difference is responsible for their varied inhibitory potential. The molecular dynamics simulations suggested that the top-ranked compounds (**3c, 3g** and **3i**) have a substantial effect on the protein dynamics which alter the protein function and have stable attachment in the protein active pocket. The findings suggest that these molecules have the potential to be investigated further as novel antidiabetic medications.

## Introduction

Diabetes mellitus (DM) has been recognized as one of the most widespread global health problems during the twenty-first century, with the prevalence surpassing 400M individuals globally^1^. According to the World Health Organization (WHO), diabetes is projected to become the seventh leading cause of death worldwide by 2030^2^. Broadly this metabolic heterogeneous disorder has two types: (i). insulin-dependent DM (type 1) and (ii). insulin-independent DM (type 2)^3^. Amidst these distinctions, both types cause alterations in glucose transport in a variety of tissue systems, leading to hyperglycemia^4^. This disruption is associated with an increased production of α-glycosidase, an enzyme responsible for hydrolysis of oligo- and disaccharides into monosaccharides^5^. Several types of α-glucosidase inhibitors have been identified to control this situation like voglibose, acarbose, and miglitol^6^, however, unfortunately, these inhibitors have shown side effects like abdominal discomfort, flatulence, and diarrhea^2^. Such complexities pose immense pressure on the pharmaceutical industry to develop novel therapeutic drugs with low toxicity and improved efficiency^7^.

**Figure 1:**
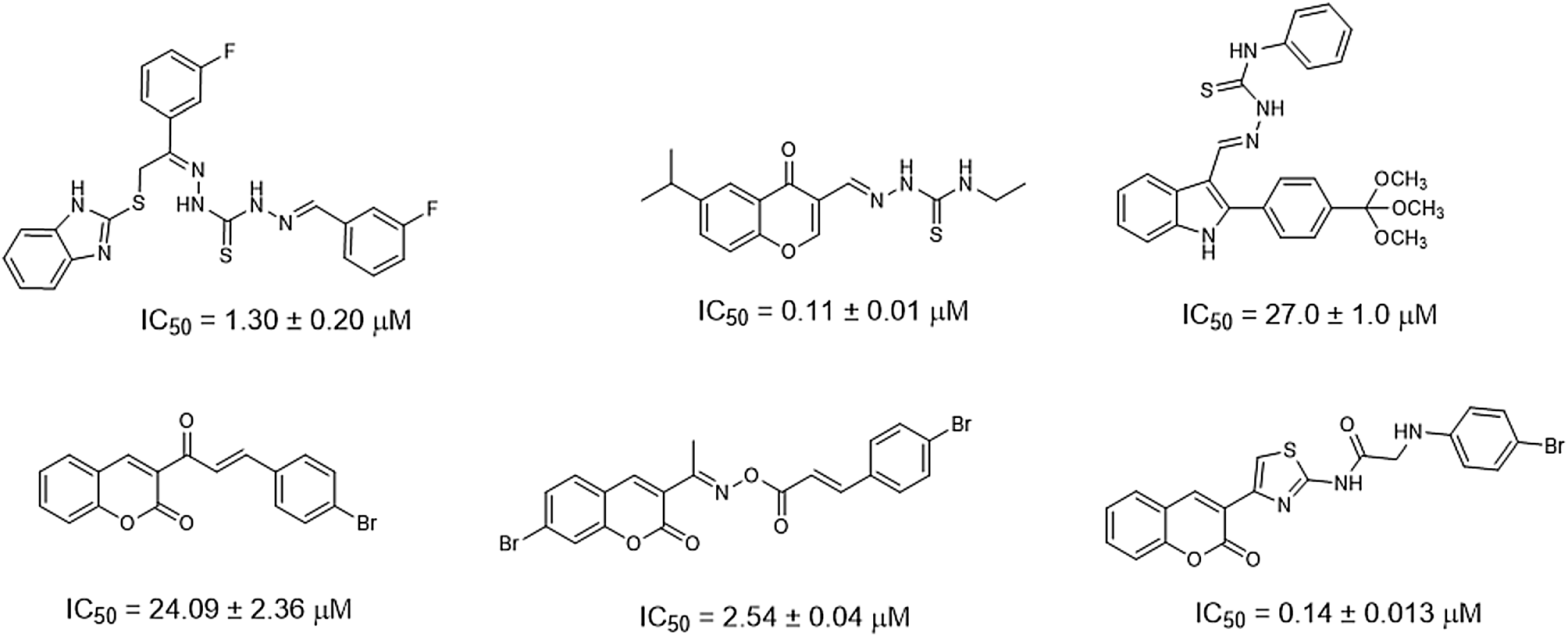
Thiosemicarbazone and Coumarin based α-glucosidase inhibitors.

Based on our thorough research, a huge number of alternative proposed compounds for α-glucosidase inhibition have been reported to date^8–11^. Lately thiosemicarbazones with general formula R^1^R^2^C=*N*–NH(C=S)NHR^12^ and typical synthetic route involving condensation of thiosemicarbazide with aldehyde/ketone have been gaining popularity among medicinal chemists^13^. These scaffolds bear a range of biological activities like antibacterial^14–16^, antifungal^17,18^, antihelmintic^19,20^, antimalarial^21,22^, antiviral^23,24^, anticancer^25,26^, and antidiabetic potential^27,28^. Additionally, several research groups have documented the comprehensive account of the antidiabetic potential of thiosemicarbazones and coumarin based compounds Fig. 1^10,29–33^.

In continuation of our efforts to develop effective α-glucosidase inhibitors^34–37^ for combating the global health condition Diabetes Mellitus (DM) and to assess the efficacy of coumarin analogues, we have designed and synthesized several coumarin derived thiosemicarbazones in this study.

## Chemistry

**Figure 2:**
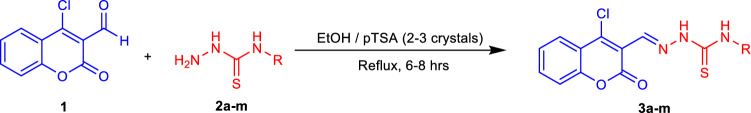
Synthetic scheme of coumarin based thiosemicarbazones **3a**–**3m**.

The preparation of coumarin derived thiosemicarbazones was carried out as in Fig. 2. Thiosemicarzides were generated by stirring the solution of phenyl isothiocyanate (5 mmol) in 20 mL of ethanol for 1 h, followed by dropwise addition of an ethanolic solution of hydrazine hydrate (5 mmol). The precipitates were filtered and washed with ethanol, and water and then dried over silica gel. Following this, thiosemicarbazone synthesis was commenced by dissolving coumarin aldehyde in EtOH at 90 °C with a few crystals of *p*-Toluenesulfonic acid (PTSA) as a catalyst. Once a clear solution is formed, respective thiosemicarbazide is introduced in the reaction mixture and set to reflux for 6–8 h. The progress of the reaction was continuously monitored through TLC. After the completion of the reaction, the reflux was stopped. Upon cooling, the coumarin derived thiosemicarbazone adduct was filtered and subsequently washed using cold MeOH.

To determine the structures of compounds **3a–3m**, spectral and elemental techniques were employed. In the infrared (IR) spectra C=S and C=N absorption bands appeared in the range of 1156–1228 cm^−1^ and 1590–1618 cm^−1^ respectively, confirming the presence of thiosemicarbazone derivatives. Likewise, C=O and NH, stretching vibrations correspondingly appeared in the region of 1699–1741 cm^−1^ and 3207–3371 cm^−1^. ^1^H NMR spectra added further structural confirmation to coumarin derived thiosemicarbazones through the observed chemical shifts for coumarin C–H and thiosemicarbazone N–H appearing within the range of 7.81–9.92 ppm and 8.74–11.19 ppm respectively. Finally, ^13^C NMR marked C=S and C=O signal at 173.52–179.38 ppm and 159.20–159.61 ppm respectively as the most downfield signals. Thus, all the analytical data provided consistent confirmation of the compound’s structures.

## Results and discussion

### In-vitro α-glucosidase inhibitory activity

In the current studies, several coumarin-derived thiosemicarbazones were synthesized and their therapeutic capability by inhibiting α-glucosidase was evaluated which can control the postprandial hyperglycemia effect of dibetes. In this context, all the synthesized compounds **3a**–**3m** were proceeded against α-glucosidase in-vitro bioassay, interestingly All of them exhibited several folds more antidiabetic potential (IC_50_ = 2.33–22.11 µM) as compared to the available marketed drug, acarbose (1C_50_ = 873.34 ± 1.67 µM). All the compounds have different R-substituents, which are responsible for the variation in the α-glucosidase inhibitory potential of these molecules. For instance, compound **3a**, with phenyl moiety exhibited potent antidiabetic activity (IC_50_ = 10.0 ± 0.14 µM), while in compound **3g**, substitution of β-phenyl increased its antidiabetic potential (IC_50_ = 2.33 ± 0.04 µM) as compared to **3a**. The methoxy group substitutions at different positions were checked in compounds **3b** and **3h**. In compound **3b**, 3-methoxyphenyl substituent resulted in potent inhibitory capability against α-glucosidase (IC_50_ = 6.10 ± 0.11 µM). While in compound **3h**, the substitution of 4-methoxyphenyl further enhanced its α-glucosidase inhibition (IC_50_ = 3.40 ± 0.08 µM) as compared to **3b**.

The effect of chlorophenyl substitution in compounds **3c** and **3k** was evaluated to identify the favorable position of substituent which is responsible for their maximum inhibitory capability. Compound **3c** with 2,4,5-trichlorophenyl group, displayed significant anti-*α*-glucosidase potential with IC_50_ of 2.57 ± 0.03 µM. On the other hand, in compound **3k**, the substitution of 2,3-dichlorophenyl declined its α-glucosidase inhibitory potential (IC_50_ = 4.22 ± 0.10 µM) as compared to **3c**. Similarly, the substitution of 2-fluorophenyl in compound **3l** further decreased the α-glucosidase inhibitory potential of **3l** with IC_50_ = 8.84 ± 0.16 µM. Similarly, we observed slight adverse effects of the substitution of benzyl and 4-tolyl groups in compounds **3d** (IC_50_ = 12.55 ± 0.16 µM) and **3e** (IC_50_ = 9.11 ± 0.20 µM), respectively. Likewise, the substitution of cyclohexyl in **3f.** produced almost similar antidiabetic effects like **3c** and **3d** with an IC_50_ value of 11.20 ± 0.24 µM.

The effect of nitrophenyl, dimethylphenyl and methyl group substitutions was investigated in compounds **3i**, **3j**, and **3m**, respectively to reveal their therapeutic use for the treatment of diabetes. In compound **3i**, the substitution of 4-nitrophenyl displayed an overwhelming anti α-glucosidase activity (IC_50_ = 2.13 ± 0.04 µM) and made it the most potent antidiabetic agent in this series. While compounds **3j** with 2,6-dimethylphenyl (IC_50_ = 22.11 ± 0.37 µM) and **3m** with single methyl substitution (IC_50_ = 24.22 ± 0.31 µM) displayed almost similar α-glucosidase inhibitory potential.

This structure–activity relationship reveals that substitution of 4-nitrophenyl, β-phenyl, and 2,4,5-trichlorophenyl in **3i**, **3g** and **3c** is most beneficial as compared to the addition of other moieties. Moreover, 3/4-methoxyphenyl in **3b**/**3h**, and 2,3-dichlorophenyl in **3k** also warrant further optimization to enhance the biological importance of **3b**, **3h**, and **3k**. The results are summarized in Table 1.

**Table 1:** A α-glucosidase inhibitory activity and R groups of compounds **3a**-**3m**.


Compounds	R	Percent inhibition (0.5 mM)	IC_50_ ± SEM (µM)
**3a**	Phenyl	92.45	10.10 ± 0.14
**3b**	3-Methoxyphenyl	94.67	6.10 ± 0.11
**3c**	2,4,5-Trichlorophenyl	94.68	2.57 ± 0.03
**3d**	Benzyl	91.20	12.55 ± 0.16
**3e**	4-Tolyl	92.75	9.11 ± 0.20
**3f**	Cyclohexyl	91.70	11.20 ± 0.24
**3g**	β-Phenyl	95.15	2.33 ± 0.04
**3h**	4-Methoxyphenyl	94.63	3.40 ± 0.08
**3i**	4-Nitrophenyl	95.30	2.13 ± 0.04
**3j**	2,6-Dimethylphenyl	91.29	22.11 ± 0.37
**3k**	2,3-Dichlorophenyl	92.54	4.22 ± 0.10
**3l**	2-Fluorophenyl	92.08	8.84 ± 0.16
**3m**	methyl	91.37	24.22 ± 0.31
Standard: acarbose	–	59.37	873.34 ± 1.67

### Kinetic studies

**Figure 3:**
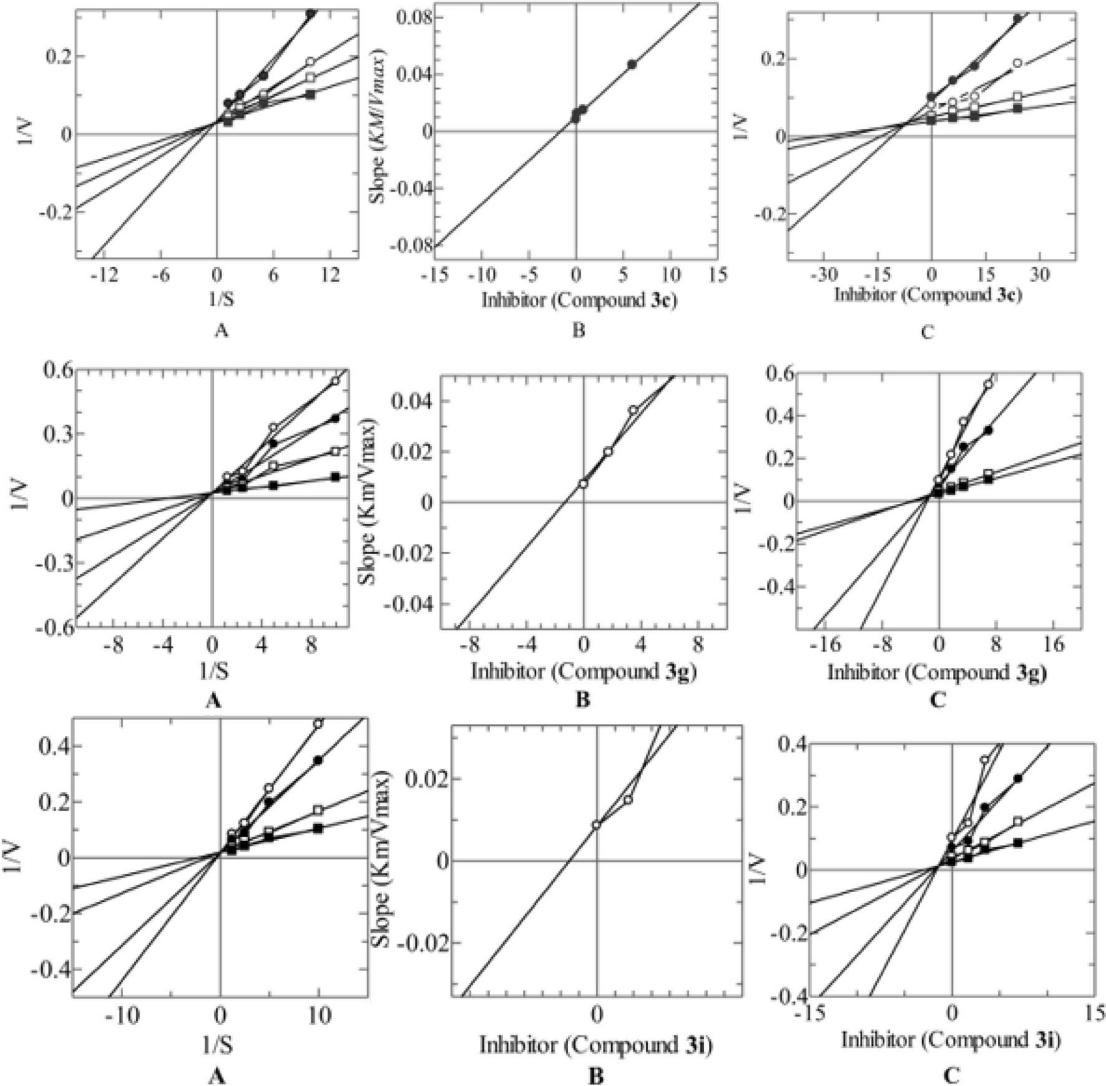
The inhibitory mode of **3c**, **3g** and **3i** against α-glucosidase enzyme (**A**) Line weaver-Burk plot of reciprocal of rate of reaction (velocities) *vs* reciprocal of substrate *p*-nitro phenyl-α-d-glucopyranoside in the absence of (filled black square), and in the presence of 7.00 (filled black circle), 3.50 (open circle) and 1.75 µM (open square) of **3c**, **3g** and **3i**. (**B**) Secondary replot of Line weaver-Burk plot between the slopes of each line on Line weaver-Burk plot against different concentrations of **3c**, **3g** and **3i**. (**C**) Dixon plot of reciprocal of rate of reaction (velocities) vs different concentrations of **3c**, **3g** and **3i**.

The most potent compounds **3c**, **3g** and **3i** were selected for kinetic studies to observe their mechanism of inhibition. In kinetic studies, compounds **3c, 3g** and **3i** were identified as the competitive inhibitor of α-glucosidase enzyme with *ki* values of 1.62 ± 0.041, 1.33 ± 0.0027 and 1.51 ± 0.0086 µM respectively. In such types of inhibition, the *Vmax* of the enzyme remains constant, while the *Km* value increases (Fig. 3). Lineweaver–Burk plots were used to determine the type of inhibition of the given compounds, in which the reciprocal of the reaction rate was plotted *vs.* the reciprocal of substrate concentrations to investigate their effect on the *Km* and *Vmax* of the α-glucosidase (Fig. 3). To determine the *Ki* values for all compounds evaluated for kinetics study secondary replot of Lineweaver–Burk plots were applied by taking the slope of each line in Lineweaver–Burk plots *vs.* different concentrations of the tested compounds. Further Dixon plots were used for the reconfirmation of *Ki* values (Fig. 3).

### Docking studies

All the compounds showed excellent inhibitory potential against the α-glucosidase enzyme; therefore, these compounds were docked at the binding site of α-glucosidase. Docking results indicate that all these molecules adopt almost the same orientation with slight conformational differences. The thiosemicarbazone moiety of these molecules is inserted deep into the active site core where this moiety has a chance to interact with any one of the residues of the catalytic triad (Asp215, Glu277, and Asp352). While the chloro-substituted coumarin ring faces the entrance of the active site, the R group has a chance to rotate towards the loop residues (Tyr72, Phe159, Phe178, Gln182, and Arg442) which lines the catalytic triad.

The most active compound **3i,** formed excellent interactions with Asp352 which is a part of the catalytic triad, in addition two crucial residues of the active site i.e., Gln353 and His112 also provide Hydrogen bonds (H-bond) to this molecule. The thiosemicarbazone moiety of **3i** mediates H-bond with the side chains of Asp352 and Gln353, while the nitro group of **3i** mediates H-bond with the side chain of His112. The thiosemicarbazone moiety of **3g**, which is the second most active compound, was oriented towards Glu277 (of the catalytic triad) and forms H-bonds with Glu277, Arg213 and His351. While the phenyl ring (R group) of **3g** interacts hydrophobically with Tyr72, and Phe178 and Phe178 provide π–π interaction to the R group. Due to the addition of bulky R groups in **3c** (tri-chlorophenyl), **3h** (methoxyphenyl) and **3k** (di-chlorophenyl), their thiosemicarbazone moiety skipped interaction with catalytic triad residues, instead **3c**, **3h** and **3k** formed H-bonds with Arg213/His351, Arg213/His112, and Arg213/His351, respectively.

**Figure 4:**
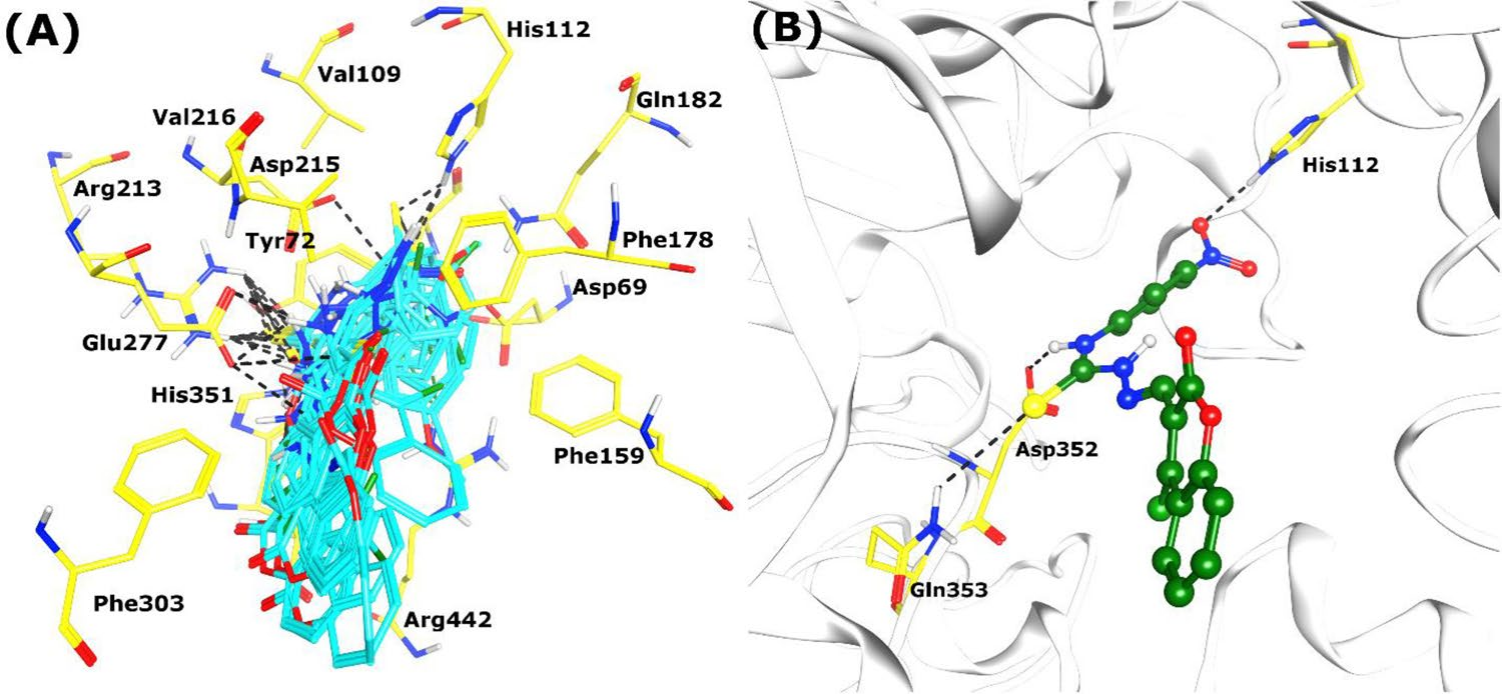
(**A**) The binding mode of all the compounds (presented in the cyan stick model) is shown in the active site of α-glucosidase. (**B**) The interactions of the most active compound (**3i**, green ball, and stick model) are shown with the active site residues (shown in yellow sticks). H-bonds are presented in black dotted lines.

Similarly, compounds **3b** and **3l** followed the interaction pattern of **3g,** and their thiosemicarbazone moiety interacted with Glu277 and Arg213. In addition, compounds **3b** and **3l** also formed H-bonds with a water molecule and His351, respectively. We observed that compounds **3e** and **3h** have almost similar binding orientation and both these molecules interacts with His112 and a water molecule. Additionally, the R group of **3e** also formed a hydrophobic (π–π) interaction with Phe303. Similarly, compounds **3a** and **3m** showed the same binding pattern, and these molecules bind with Asp352, and His351 through H-bonds. While the thiosemicarbazone functional group of **3f** does not contribute to the binding interaction, and only its coumarin mediates H-bonding with the side chain of Gln353, and forms π-π interactions with Phe303. Likewise, the thiol group of **3d** only formed an H-bond with His112. Like **3f**, the coumarin oxygen of **3j** also interacted with Gln353, while the carbazide nitrogen of this molecule formed an H-bond with the side chain of Asp352 like compounds **3a** and **3m**. The docking results indicate that the conformational difference can affect the binding pattern, as a result, the activity is affected. The docking scores and interactions are given in Table 2. The docking scores of the compounds are in the range of − 6.34 kcal/mol to − 3.21 kcal/mol, which reflects the good binding potential of these molecules with the active site of the enzyme. The binding modes of compounds are depicted in Fig. 4. The most active compounds (**3i**, **3g**, and **3c**) exhibited the highest docking scores, i.e., − 6.34 to − 6.34 kcal/mol, likewise, the moderate active molecules demonstrated docking scores in range of > − 5 to > − 3 kcal/mol, and two least active molecules in this series (**3j** and **3m**) bear the least docking scores. According to the docking scores of compounds, the docking results correlate well with the in-vitro assay results. Furthermore, docking analysis reflects the importance of Asp352, Gln353, His112, Glu277, Arg213, His351, and Arg442 in the stabilization of compounds in the binding site of the enzymes, while Phe178, and Phe303 are crucial to providing hydrophobic interactions to these novel inhibitors.

**Table 2:** The docking scores and interactions of compounds **3a**-**3m** with the α-glucosidase.

Compounds	Docking score (kcal/mol)	Ligand atom	Receptor atom	Interactions	Distance (Å)
**3i**	− 6.77	N5	OD2-ASP352	HBD	2.02
		S7	NE2-GLN353	HBA	3.38
		O19	NE2-HIS112	HBA	2.38
**3g**	− 6.60	N1	OE2-GLU277	HBD	2.52
		S7	NH1-ARG213	HBA	3.48
		S7	NH2-ARG213	HBA	3.18
		S7	NE2-HIS351	HBA	3.13
		6-ring	6-ring-PHE178	π-π	3.69
**3c**	− 6.34	S7	NH1-ARG213	HBA	3.02
		S7	NH2-ARG213	HBA	2.53
		S7	NE2-HIS351	HBA	2.91
**3h**	− 5.25	S7	NE2-HIS112	HBA	2.83
		S7	O-HOH803	HBA	3.13
		O39	NH2-ARG213	HBA	2.12
**3k**	− 5.81	S7	NH1-ARG213	HBA	3.26
		S7	NH2-ARG213	HBA	3.14
		S7	NE2-HIS351	HBA	3.86
**3b**	− 5.31	N1	OE1-GLU277	HBD	2.96
		N3	O-HOH986	HBA	3.31
		S7	NH1-ARG213	HBA	2394
		S7	NH2-ARG213	HBA	2.88
**3l**	− 4.91	N1	OE1-GLU277	HBD	3.10
		N1	OE2-GLU277	HBD	3.16
		S7	NH1-ARG213	HBA	3.15
		S7	NH2-ARG213	HBA	2.76
		S7	NE2-HIS351	HBA	2.83
**3e**	− 4.72	S7	NE2-HIS112	HBA	2.86
		S7	O-HOH803	HBA	3.22
		6-ring	6-ring-PHE303	π-π	3.96
**3a**	− 4.44	N1	OD2-ASP352	HBD	1.49
		S7	NE2-HIS351	HBA	3.09
**3f**	− 4.04	O41	NE2-GLN353	HBA	2.72
		6-ring	6-ring-PHE303	π-π	3.97
**3d**	− 3.80	S7	NE2-HIS112	HBA	3.12
**3j**	− 3.28	N1	OD2-ASP352	HBD	2.76
		O41	NE2-GLN353	HBA	3.04
		6-ring	NH1-ARG442	π-cation	3.44
**3m**	− 3.21	N1	OD2-ASP352	HBD	2.19
		S7	NE2-HIS351	HBA	2.51

*HBA* hydrogen bond acceptor, *HBD* hydrogen bond donor.

### Post-simulation analysis

#### Examining the protein dynamic stability

**Figure 5:**
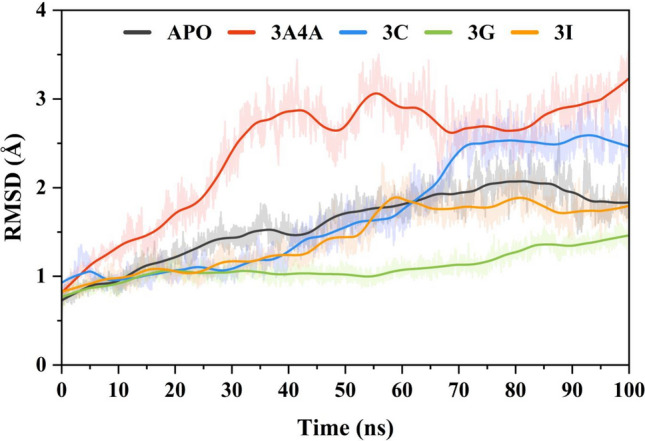
Root mean square deviation analysis of the α-glucosidase in free state reference inhibitor attached (3A4A) and selected compounds (**3c**, **3g**, and **3i**) from 100 ns simulation trajectories.

The RMSD of a simulation snapshot indicates how closely it matches a reference structure, which might be the starting simulation frame or a crystal structure. This parameter is used to examine the development of structural elements over time [70]. It is particularly useful in determining if a structure remains stable during the simulation time or deviates from its beginning coordinates. To assess the protein's stability during the simulation, we examined the RMSD plot of the Cα atoms (Fig. 5). This included comparing each frame in time to the simulation's initial frame. The RMSDs for the apo form of -glucosidase, the -glucosidase-maltose complex (3A4A positive control), and three -glucosidase -ligand complexes produced by molecular docking were calculated. The APO protein average RMSD calculated was 1.59 ± 0.003 Å. During the initial 60 ns, the RMSD of the protein gradually increases from 0.6 to 1.8 Å. The RMSD then remains stable for the rest of the simulation time. On the other hand, the ligand-bound protein 3A4A shows a significant increase in the mean RMSD (2.42 ± 0.006 Å). A rapid increase was observed in the first 40 ns simulation time (2 Å increase), where the protein went through structure confirmation from 41 to 70 ns simulation time. After 70 ns, the system got equilibrium and retained a stable RMSD with an average value of 2.7 Å. In the selected compounds complexes, **3c** shows an average RMSD of 1.69 ± 0.006 Å, while the **3g** and **3i** mean RMSD calculated was 0.01 ± 0.001 Å and 1.43 ± 0.003 Å, respectively. The **3c** system shows flexibility in the start 70 ns simulations where the RMSD value was increased gradually (0.9 Å to 2.4 Å). After that, the system gains equilibrium and has a stable RMSD value (2.5 Å mean RMSD). Compound **3g** retains a stable behavior in the RMSD pattern till the end of the simulation, where the first 20 ns shows a slight increase in the RMSD values. The RMSD values of the **3i** system increased from the start till 60 ns with a gradual increase pattern (1 Å increased). The system retains a stable RMSD by gaining equilibrium till the end of the simulation (1.8 Å mean RMSD). The RMSD results indicate that the ligand-attached systems showed a stable complex by retaining a stable RMSD for most of the simulation time. Thus, it signifies that the ligand and the target protein have a favorable interaction because the unbound protein may naturally adopt a conformation that favors binding with the small molecule in the complex. Further, the RMSD results indicate that there were no significant jumps in each system's RMSD profile, which signifies the simulations' output results.

#### Residues fluctuations measurement

**Figure 6:**
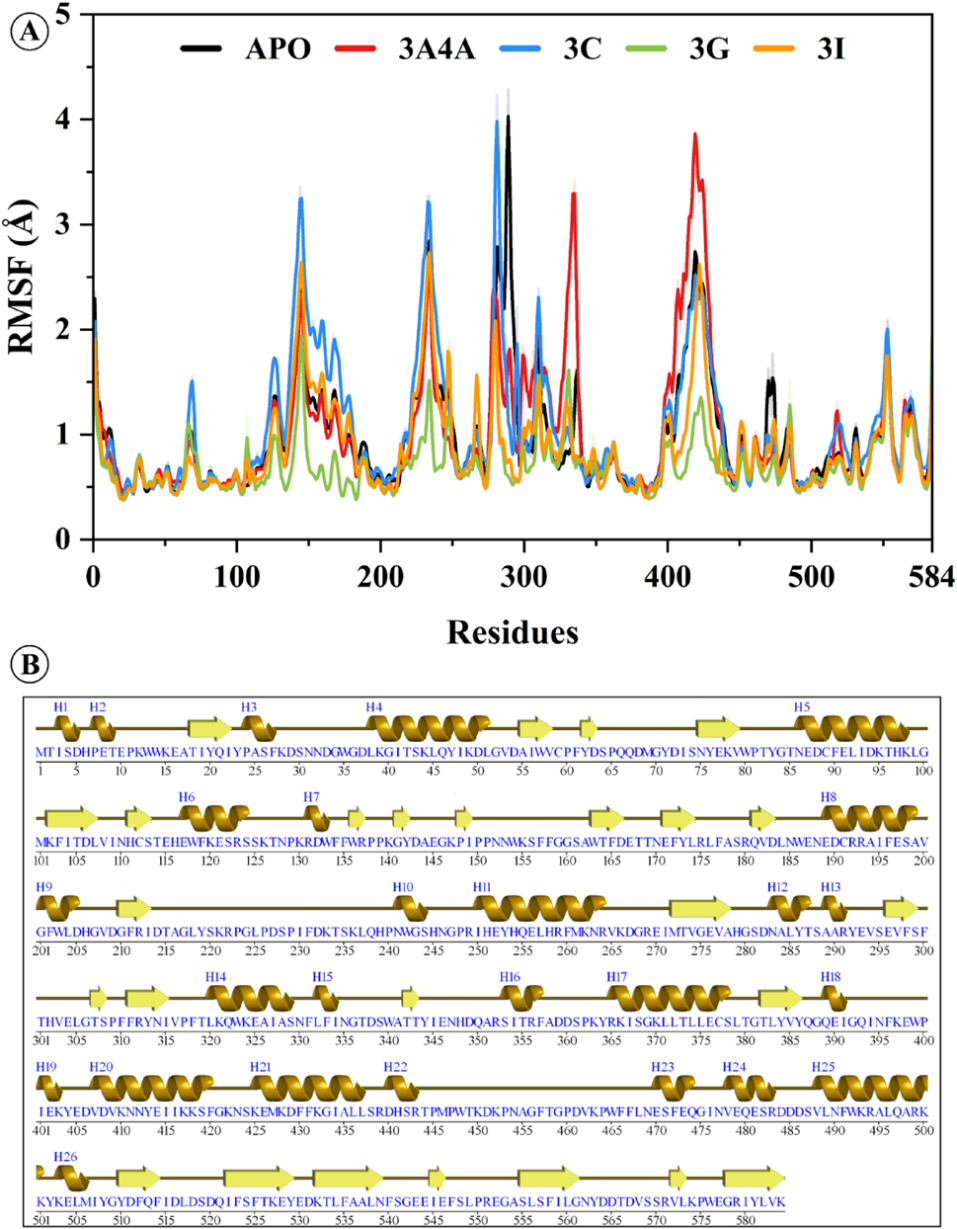
Root mean square fluctuation analysis to analyze the residues flexibility of the selected compound with a reference system 3A4A and free state in 100 ns simulation time.

Following that, we investigated whether protein residues or segments contributed to the observed changes in mobility shown in the RMSF data (Fig. 6). The RMSF value is critical for understanding how the side chains of residues change during MD simulation. The average RMSF calculated for the APO protein was 0.97 ± 0.02 Å. The high mobility regions observed in the APO protein were 276–298, 216–252, and 394–442 residues. The residues regions 276–298 and 216–252 show a loop confirmation, while the 394–442 region has a loop and H-19 to H22 helix regions. The average RMSF calculated for the reference system 3A4A was 1 ± 0.02 Å. The 394–442 residues in the 3A4A show a considerable fluctuation exceeding the 2 Å. The residues region 270–340 shows a confirmational shift from the free state protein, where the protein went structural confirmation. The average RMSF of the **3c** system was 1.1 ± 0.02 Å, where the residues region 122–182 show a variable structural confirmation which exceeds the 2 Å value. The structural confirmation of the 122–182 residues region shows that most of the region is loop confirmation, which shows flexibility in the simulation. The **3g** and **3i** systems show mean RMSF values of 0.7 ± 0.01 Å and 0.8 ± 0.01 Å, respectively. The **3g** system shows restricted movements in the regions that fluctuate high (above 2 Å) in the APO system, and the **3g** shows fluctuation below 2 Å in the overall simulation. Similarly, the **3i** system retains a stable RMSF where the 132–182 and 219–250 residues region shows high flexibility. The residues 376–312 region shows a restricted motion compared to the APO, where the APO shows fluctuation up to 4 Å. The RMSF results show that the protein adopts structural confirmation upon the ligand attachment with the protein active pocket. This indicates that the selected compounds bind tightly to the protein active site residues. The overall RMSF results of each system indicate that the proteins remain stable during the simulation run.

#### Protein compactness analysis

**Figure 7:**
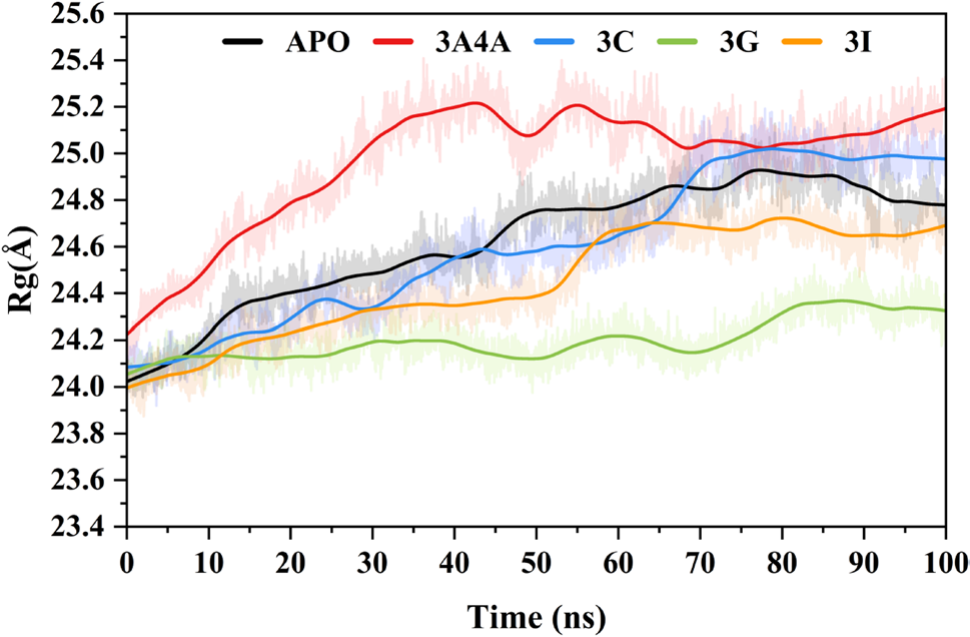
Protein compactness analysis of selected compounds free state (APO) and reference inhibited system 3A4A via Radius f gyration.

The RG values calculated from MD modeling of unbound proteins and protein–ligand complexes may be regarded as measures of their overall compactness. It represents the root mean square distance between the component atoms' centers of mass. A lower RG number indicates a more compact and firmly folded structure, while a larger value indicates a more extended or flexible conformation. The value aids in determining how ligand binding affects the overall shape and flexibility of the protein by giving insight into the structural dynamics and stability of the protein or protein–ligand complex. Figure 7 shows the calculated RG profiles of simulated systems. The average Rg calculated for the APO protein was 24.62 ± 0.002 Å. In the first 60 ns simulation time, the Rg of the APO system increases gradually, and after 70 ns, the system gets a stable Rg having a compact protein confirmation. The reference ligand attached to system 3A4A shows a mean Rg value of 24.97 ± 0.002 Å. The 3A4A system shows flexibility in the first 50 ns simulation time where the Rg increases (0.9 Å increase). After that, the system retains the Rg with a mean value of 25.05 Å. A steady increase of 1.8 Å from the initial values to 70 ns simulation time. The system retains the Rg with a mean value of 24.9 Å till the end of the simulation. The average Rg values of the **3c** and **3g** systems are 24.60 ± 0.03 Å and 24.19 ± 0.001 Å, respectively. The **3g** system retains a stable Rg in the simulation with minor bumps. The average Rg calculated for the **3i** system reported was 24.44 ± 0.002 Å. The first 50 ns simulation time shows a constant increase in the Rg value (24.0 Å to 24.6 Å). After that, the structural confirmation of the system's Rg remains steady until the end of the simulation (average Rg 24.7 Å). As per the Rg results, the protein experienced conformational changes during the initial of the simulation to better constrain the ligand within the pocket, followed by establishing solid contact with the ligand and obtaining a stable Rg.

#### Protein and ligand binding affinity estimation

The BFE was calculated using the most stable output MD trajectories (the final 2750 frames of each complex) of the three α-glucosidase-inhibitor structures and a reference 3A4A system using the MM-GBSA technique. The MM-GB/PBSA method uses a continuum solvent model to calculate the complex's binding free energy values. As a result, the BFE values determined in this research are related to the absolute or total free energy. The complexes with the lowest BFE value were the most stable. Table 3 contains the computed values. The results revealed variations in the BFE among the ligands. The reference compound 3A4A showed a stable complex with a total BFE (ΔG_TOTAL_) value of -12.8 ± 0.10 kcal/mol. Further analysis showed that contributions of electrostatic energy (ΔEELE) and gas phase free energy (ΔGgas) were the most significant factors in binding this ligand to the protein. Based on the results from the MMGBSA calculation, the **3g** compound exhibits the highest BFE of − 24.7 ± 0.07 kcal/mol. The second highest binding energy was reported for the **3i** compound of − 22.7 ± 0.07 kcal/mol, while the **3c** system calculated binding energy is − 17.2 ± 0.06 kcal/mol. Consequently, the selected compounds (**3c**, **3g,** and **3i**) complexes are likely more stable than the reference compound 3A4A. The **3c** system ΔE_ELE_ and ΔG_gas_ components are the main contributors to the BFE, while the **3g** and **3i** system shows reduced in the ΔE_ELE_ and high ΔE_VDW_ contribution. In conclusion, the study's results show that the selected compounds can serve as α-glucosidase inhibitors, making them viable candidates for building putative antidiabetic medicines.

**Table 3:** Ligand binding free energies computed using the MM-GBSA method: calculations done using 2750 complex frames.

Complex	ΔE_VDW_	ΔE_ELE_	ΔE_GB_	ΔE_SURF_	ΔG_solv_	ΔG_gas_	ΔG _TOTAL_
**3A4A**	− 30.6 ± 0.08	− 205.0 ± 0.55	228.27 ± 0.47	− 5.3 ± 0.011	222.8 ± 0.47	− 235.7 ± 0.54	− 12.8 ± 0.10
**3c**	− 24.9 ± 0.07	− 186.1 ± 0.25	197.1 ± 0.24	− 3.3 ± 0.006	193.8 ± 0.24	− 211.0 ± 0.26	− 17.2 ± 0.06
**3g**	− 39.9 ± 0.07	− 21.1 ± 0.13	41.4 ± 0.12	− 5.0 ± 0.007	36.3 ± 0.12	− 61.1 ± 0.13	− 24.7 ± 0.07
**3i**	− 43.8 ± 0.07	− 16.0 ± 0.15	42.6 ± 0.17	− 5.5 ± 0.006	37.1 ± 0.17	− 59.8 ± 0.18	− 22.7 ± 0.07

All measurements are in kcal/mole. The numbers presented are the mean values for the complex obtained after subtracting the ligand and receptor energy from the complex energy. The distinction is complex–ligand–receptor.

ΔE_VDW_, van der Waals free energy: ΔE_ELE_, electrostatic free energy: ΔE_GB_, the polar component of solvation-free energy calculated via Generalized-Born method: ΔE_SURF_, the non-polar component of the solvation energy computed via SASA: ΔG_gas_, gas phase energy: ΔG_TOTAL_, total binding free energy.

## Experimental

### Material

All chemical reagents were purchased from Sigma-Aldrich Company, and no additional purification was performed. Commercially available oven-dried round bottom flasks were utilized for the reaction. The Heraeus Elemental Analyzer was used to execute elemental analyses. On an ABB Bomem FTIR instrument, KBr pellets were used to record infrared spectra in the range of 4000–500 cm^−1^. The Bruker DRX 500 with TMS as standard and DMSO-d^6^ solvent was used to record the ^1^H NMR and^13^C NMR spectra.

#### (E)-2-((4-chloro-2-oxo-2H-chromen-3-yl)methylene)-N-phenylhydrazine-1-carbothioamide (3a)

Yellowish solid, Yield, 86%; IR υ_max_ (cm^−1^): 1623 (C=N), 1655 (C=O), 3365 (N–H); ^1^H NMR (400 MHz, DMSO-d_6_) δ, ppm 12.29 (1 H, s), 9.76 (1 H, s), 8.39 (1 H, s), 8.02 (1 H, dd, *J* 8.2, 1.3), 7.79–7.72 (1 H, m), 7.68 (2 H, d, *J* 7.6), 7.54–7.47 (2 H, m), 7.39 (2 H, dd, *J* 10.8, 5.0), 7.21 (1 H, t, *J* 7.4).^13^C NMR (101 MHz, DMSO) δ 176.10, 157.80, 151.68, 145.72, 138.93, 136.52, 134.26, 128.91, 126.45, 125.90, 125.77, 124.39, 119.32, 118.80, 117.07; Anal. Calcd. for C_17_H_12_ClN_3_O_2_S (357.81): C, 57.07; H, 3.38; N, 11.74; Found: C, 57.11; H, 3,33; N, 11.89.

#### (E)-2-((4-chloro-2-oxo-2H-chromen-3-yl)methylene)-N-(3-methoxyphenyl)hydrazine-1-carbothioamide (3b)

Yellowish solid, Yield, 82%; IR υ_max_ (cm^−1^): 1631 (C=N), 1666 (C=O), 3340 (N–H); ^1^H NMR (400 MHz, DMSO-d_6_) δ, ppm 12.31 (1 H, s), 9.75 (1 H, s), 8.38 (1 H, s), 8.03 (1 H, dd, *J* 8.3, 1.4), 7.80–7.72 (1 H, m), 7.54–7.45 (3 H, m), 7.29 (1 H, t, *J* 8.1), 7.22–7.14 (1 H, m), 6.82–6.74 (1 H, m), 3.77 (3 H, s); ^13^C NMR (101 MHz, DMSO) δ 175.79, 159.70, 157.77, 151.69, 145.76, 140.04, 136.49, 134.28, 129.71, 126.46, 125.91, 119.32, 118.80, 117.07, 116.27, 111.14, 109.77, 55.65; Anal calcd for C_18_H_14_ClN_3_O_3_S (387.84); C, 55.74; H, 3.64; 10.83; Found: C, 55.91; H, 3.77; 10.97.

#### (E)-2-((4-chloro-2-oxo-2H-chromen-3-yl)methylene)-N-(2,4,5-trichlorophenyl) hydrazine-1-carbothioamide (3c)

Off-white solid, Yield, 90%; IR υ_max_ (cm^−1^): 1639 (C=N), 1664 (C=O), 3354 (N–H); ^1^H NMR (400 MHz, DMSO-d_6_) δ, ppm 12.64 (1 H, s), 9.83 (1 H, s), 8.56 (1 H, s), 8.45 (1 H, s), 8.05 (1 H, dd, *J* 8.3, 1.5), 7.99 (1 H, s), 7.81–7.72 (1 H, m), 7.51 (2 H, ddd, *J* 5.9, 3.7, 1.3); ^13^C NMR (101 MHz, DMSO) δ 176.17, 157.82, 151.81, 146.14, 138.25, 136.19, 134.44, 130.79, 129.82, 128.86, 128.06, 127.87, 126.62, 125.92, 118.99, 118.81, 117.09; Anal calcd for C_17_H_9_Cl_4_N_3_O_2_S (461.14); C, 44.28; H, 1.97; N, 9.11; Found: C, 44.36; H, 2.01; N, 9.33.

#### (E)-N-benzyl-2-((4-chloro-2-oxo-2H-chromen-3-yl) methylene) hydrazine-1-carbothioamide (3d)

Light Yellow solid, Yield, 88%; IR υ_max_ (cm^−1^): 1641 (C=N), 1654 (C=O), 3334 (N–H); ^1^H NMR (400 MHz, DMSO-d_6_) δ, ppm 12.05 (1 H, s), 8.45 (1 H, t, *J* 6.1), 8.30 (1 H, d, *J* 0.7), 8.00 (1 H, dd, *J* 8.3, 1.4), 7.77–7.69 (1 H, m), 7.48 (2 H, ddd, *J* 4.4, 3.6, 1.9), 7.35 (4 H, t, *J* 5.5), 7.31–7.22 (1 H, m), 4.86 (2 H, d, *J* 6.1); ^13^C NMR (101 MHz, DMSO) δ 178.24, 157.99, 151.62, 145.07, 139.25, 136.34, 134.08, 128.89, 128.81, 128.64, 128.26, 127.94, 127.70, 127.45, 126.42, 125.84, 119.44, 118.83, 117.02, 47.25.

Anal calcd for C_18_H_14_ClN_3_O_2_S (371.84); C, 58.14; H, 3.80; N, 11.30; Found: C, 58.24; H, 3.77; N, 11.52.

#### (E)-2-((4-chloro-2-oxo-2H-chromen-3-yl) methylene)-N-(p-tolyl) hydrazine-1-carbothioamide (3e)

Yellow solid, Yield, 96%; IR υ_max_ (cm^−1^): 1654 (C=N), 1655 (C=O), 3342 (N–H); ^1^H NMR (400 MHz, DMSO-d_6_) δ, ppm 12.25 (1 H, s), 9.67 (1 H, s), 8.37 (1 H, s), 8.02 (1 H, dd, *J* 8.3, 1.4), 7.81–7.71 (1 H, m), 7.51 (4 H, ddd, *J* 6.3, 5.3, 3.4), 7.18 (2 H, d, *J* 8.2), 2.31 (3 H, s); ^13^C NMR (101 MHz, DMSO) δ 176.10, 157.80, 151.67, 145.62, 136.38, 135.03, 134.23, 129.51, 129.33, 126.44, 125.89, 124.46, 119.35, 118.81, 117.06, 21.03; Anal calcd for C_18_H_14_ClN_3_O_2_S (371.84); C, 58.14; H, 3.80; N, 11.30; Found: C, 58.29; H, 3.71; N, 11.60.

#### (E)-2-((4-chloro-2-oxo-2H-chromen-3-yl)methylene)-N-cyclohexylhydrazine-1-carbothioamide (3f)

White solid, Yield, 84%; IR υ_max_ (cm^−1^): 1651 (C=N), 1650 (C=O), 3335 (N–H); ^1^H NMR (400 MHz, DMSO-d_6_) δ, ppm 11.95 (1 H, s), 8.29 (1 H, d, *J* 0.7), 8.04–7.99 (1 H, m), 7.77–7.71 (2 H, m), 7.53–7.46 (2 H, m), 4.22–4.07 (1 H, m), 1.91 (2 H, t, *J* 8.7), 1.67 (2 H, s), 1.56 (1 H, d, *J* 12.0), 1.37 (4 H, dd, *J* 18.8, 9.9), 1.27 (1 H, d, *J* 9.2); ^13^C NMR (101 MHz, DMSO) δ 176.52, 157.73, 151.59, 144.87, 135.56, 134.08, 126.35, 125.84, 119.43, 118.84, 117.02, 52.04, 32.16, 25.49, 24.58; Anal calcd for C_17_H_18_ClN_3_O_2_S (363.86); C, 56.12; H, 4.99; N, 11.55; Found: C, 56.19; H, 5.09; N, 11.67.

#### (E)-2-((4-chloro-2-oxo-2H-chromen-3-yl) methylene)-N-phenethylhydrazine-1-carbothioamide (3g)

Yellow solid, Yield, 87%; IR υ_max_ (cm^−1^): 1655 (C=N), 1641 (C=O), 3344 (N–H); ^1^H NMR (400 MHz, DMSO-d_6_) δ, ppm 11.95 (1 H, s), 8.26 (1 H, s), 8.00 (1 H, dd, *J* 8.3, 1.1), 7.95 (1 H, t, *J* 5.6), 7.74 (1 H, td, *J* 8.0, 1.5), 7.51 (2 H, dd, *J* 11.9, 4.4), 7.38–7.21 (5 H, m), 3.83 (2 H, dd, *J* 13.3, 7.0), 2.92 (2 H, t, *J* 7.3); ^13^C NMR (101 MHz, DMSO) δ 177.70, 158.16, 151.59, 144.72, 139.43, 136.11, 134.04, 129.11, 128.98, 126.77, 126.42, 125.87, 119.35, 118.89, 117.04, 45.33, 34.92; Anal calcd for C_19_H_16_ClN_3_O_2_S (385.87); C, 59.14; H, 4.18; N, 10.89; Found: C, 59.19; H, 4.31; N, 10.99.

#### (E)-2-((4-chloro-2-oxo-2H-chromen-3-yl) methylene)-N-(4-methoxyphenyl) hydrazine-1-carbothioamide (3h)

Light yellow solid, Yield, 80%; IR υ_max_ (cm^−1^): 1645 (C=N), 1636 (C=O), 3321 (N–H); ^1^H NMR (400 MHz, DMSO-d_6_) δ, ppm 12.22 (1 H, s), 9.60 (1 H, s), 8.37 (1 H, s), 8.03 (1 H, dd, *J* 8.3, 1.5), 7.80–7.72 (1 H, m), 7.56–7.46 (4 H, m), 6.96–6.90 (2 H, m), 3.77 (3 H, s); ^13^C NMR (101 MHz, DMSO) δ 176.50, 157.88, 157.42, 151.67, 145.56, 136.38, 134.21, 131.84, 126.61, 126.45, 125.90, 119.39, 118.82, 117.07, 114.03, 55.75; Anal calcd for C_18_H_14_ClN_3_O_3_S (387.84); C, 55.74; H, 3.64; N, 10.83; Found: C, 55.91; H, 3.52; N, 10.96.

#### (E)-2-((4-chloro-2-oxo-2H-chromen-3-yl) methylene)-N-(4-nitrophenyl) hydrazine-1-carbothioamide (3i)

Yellow solid, Yield, 90%; IR υ_max_ (cm^−1^): 1647 (C=N), 1655 (C=O), 3342 (N–H); ^1^H NMR (400 MHz, DMSO-d_6_) δ, ppm 10.44 (1 H, s), 8.47 (1 H, s), 8.23–8.16 (3 H, m), 7.89–7.84 (2 H, m), 7.80–7.74 (1 H, m), 7.56–7.47 (2 H, m); ^13^C NMR (101 MHz, DMSO) δ 157.97, 153.66, 153.16, 149.78, 146.60, 142.03, 134.89, 134.58, 127.24, 125.52, 125.41, 121.15, 119.47, 118.80, 117.43, 99.99; Anal calcd for C_17_H_11_ClN_4_O_4_S (402.81); C, 50.69; H, 2.75; N, 13.91; Found: C, 50.77; H, 2.71; N, 13.96.

#### (E)-2-((4-chloro-2-oxo-2H-chromen-3-yl) methylene)-N-(2,6-dimethylphenyl) hydrazine-1-carbothioamide (3j)

Yellow solid, Yield, 88%; IR υ_max_ (cm^−1^): 1641 (C=N), 1625 (C=O), 3331 (N–H); ^1^H NMR (400 MHz, DMSO-d_6_) δ, ppm 12.19 (1 H, s), 9.30 (1 H, s), 8.36 (1 H, s), 8.02 (1 H, dd, *J* 8.4, 1.5), 7.78–7.71 (1 H, m), 7.54–7.47 (2 H, m), 7.14–7.07 (3 H, m), 2.18 (6 H, s); ^13^C NMR (101 MHz, DMSO) δ 177.59, 158.31, 151.64, 145.17, 137.14, 136.77, 136.52, 134.08, 128.15, 127.58, 126.50, 125.88, 119.41, 118.94, 117.05, 18.46; Anal calcd for C_19_H_16_ClN_3_O_2_S (385.87); C, 59.14; H, 4.18; N, 10.89; Found: C, 59.21; H, 4.09; N, 10.99.

#### (E)-2-((4-chloro-2-oxo-2H-chromen-3-yl) methylene)-N-(2,3-dichlorophenyl) hydrazine-1-carbothioamide (3k)

Off-white solid, Yield, 92%; IR υ_max_ (cm^−1^): 1633 (C=N), 1635 (C=O), 3345 (N–H); ^1^H NMR (400 MHz, DMSO-d_6_) δ, ppm 12.54 (1 H, s), 9.82 (1 H, s), 8.44 (1 H, s), 8.05 (2 H, ddd, *J* 8.2, 3.8, 1.3), 7.81–7.72 (1 H, m), 7.58–7.48 (3 H, m), 7.42 (1 H, t, *J* 8.1); ^13^C NMR (101 MHz, DMSO) δ 176.60, 158.00, 151.77, 145.82, 138.13, 137.83, 134.35, 132.09, 128.14, 128.08, 127.61, 127.02, 126.59, 125.91, 119.09, 118.86, 117.09; Anal calcd for C_17_H_10_Cl_3_N_3_O_2_S (426.70); C, 47.85; H, 2.36; N, 9.85; Found: C, 47.90; H, 2.33; N, 9.99.

#### (E)-2-((4-chloro-2-oxo-2H-chromen-3-yl) methylene)-N-(2-fluorophenyl) hydrazine-1-carbothioamide (3l)

White solid, Yield, 81%; IR υ_max_ (cm^−1^): 1643 (C=N), 1636 (C=O), 3351 (N–H); ^1^H NMR (600 MHz, DMSO-d_6_) δ, ppm 12.44 (1 H, s), 9.61 (1 H, s), 8.40 (1 H, s), 8.09–8.00 (2 H, m), 7.74 (1 H, dd, *J* 11.4, 4.0), 7.49 (2 H, t, *J* 7.1), 7.29 (2 H, dd, *J* 19.9, 9.0), 7.22 (1 H, t, *J* 7.4); ^13^C NMR (151 MHz, DMSO) δ 176.31, 157.58, 156.36, 154.73, 151.27, 145.11, 136.87, 133.82, 127.22, 127.16, 126.95, 126.69, 126.62, 126.09, 125.45, 124.05, 118.71, 118.42, 116.62, 115.59, 115.46; Anal calcd for C_17_H_11_ClFN_3_O_2_S (375.80); C, 54.33; H, 2.95; N, 11.18; Found: C, 54.44; H, 2.83; N, 11.31.

#### (E)-2-((4-chloro-2-oxo-2H-chromen-3-yl) methylene)-N-methylhydrazine-1-carbothioamide (3m)

Light yellow solid, Yield, 87%; IR υ_max_ (cm^−1^): 1652 (C=N), 1654 (C=O), 3332 (N–H); ^1^H NMR (600 MHz, DMSO-d_6_) δ, ppm 11.88 (1 H, s), 8.25 (1 H, s), 8.00 (2 H, d, *J* 8.0), 7.73 (1 H, t, *J* 7.5), 7.49 (2 H, t, *J* 7.1), 3.04 (3 H, d, *J* 4.5); ^13^C NMR (151 MHz, DMSO) δ 178.06, 157.67, 151.16, 144.47, 135.55, 133.58, 125.97, 125.41, 119.03, 118.42, 116.59, 31.12; Anal calcd for C_12_H_10_ClN_3_O_2_S (295.74); C, 48.74; H, 3.41; N, 14.21; Found: C, 48.87; H, 3.35; N, 14.31.

### In-vitro α-glucosidase assay

The most recent investigations were carried out utilizing the techniques we previously discussed^38^. The enzyme and substrate were dissolved in phosphate buffer (pH 6.8) which also served as the reaction buffer for the experiments. A 96-well plate was used for the following ingredients: enzyme 2 U/2 mL, test samples (0.5 mM), 20 L/well, enzyme 20 L/well, and 135 L/well reaction buffer. The plate was incubated for 15 min at 37 °C. Changes in absorbance brought on by substrate breakdown were measured at 400 nm for 30 min after each well-received 25 L of 4-nitro phenyl *α*-D-glucopyranoside. Acarbose served as a positive control, and 7% DMSO served as a negative control.

### Statistical analysis

The results of the recent research on the anti-diabetic impact of the studied materials presented were obtained using Excel and the SoftMax Pro suite.

The formula below was used to compute the percent inhibition of all drugs tested.

1 $$the \begin{array}{c}\%Inhibition=100-\left(\frac{{O.D}_{test \; compound}}{{O.D}_{control}}\right)\times 100\end{array}$$

EZ-FIT (Perrella Scientific, Inc., USA) to calculate the IC_50_ for all antidiabetic agents under the study. All experiments were carried out in triplicate to prevent predicted errors, and variances in results are reported as Standard Error of Mean values (SEM).

2 $$\begin{array}{c}SE=\frac{\sigma }{\sqrt{n}}\end{array}$$

### Molecular docking

Docking of all the compounds was performed by Molecular Operating Environment (MOE version 2022.02)^39,40^. For the in-silico experiment, the X-ray crystal structure of α-glucosidase from an inbound form with α-D-glucopyranose was selected (PDB code 3A4A)^38^. The structures of compounds were first drawn on the Chem Draw program and saved into mol format, then each structure was imported into the MOE compound’s database, where the MOE WASH module was used to convert the structures 3D-format. During this conversion, Hydrogen atoms were added simultaneously on ligands, and partial charges (MMFF94x force field) were calculated. Later the structures of ligands were energy minimized with a gradient of 0.1kcal/mol/Å. The α-glucosidase enzyme file was modified for docking by adding hydrogen atoms and calculating charges (with AMBER 10:EHT force field) on residues through the QuickPrep module of MOE. Subsequently, docking was accomplished with default docking parameters of MOE, i.e., Triangle Matcher placement method and London dG scoring function. This docking procedure was initially validated by re-docking of co-crystallized ligand (isomaltose) in its original position in the PDB file, which perfectly bind at its cognate position determined by the X-ray crystallography with RMSD of 0.16 Å (Fig. S1, supporting information). In the end, thirty conformations of each ligand were saved for further analysis.

### Molecular dynamics simulations

In addition to providing energetic insights into protein–ligand interactions, molecular dynamics (MD) simulation can give various dynamic structural information regarding biomacromolecules. These discoveries might lead to more effective strategies for creating protein–ligand interactions. To properly guide the drug development process, a solid knowledge of protein–ligand interactions is crucial^41^. The conformational dynamics of numerous protein systems were investigated in this work using MD simulation. These systems included apo α-glucosidase, three highly rated α-glucosidase-inhibitor complexes (**3c**, **3g**, and **3i**), and a reference complex (α-glucosidase-alpha-d-glucopyranose complex, PDB ID: 3A4A). The AMBER22^2^ software with an explicit solvent model was used during the simulation. The simulation was initiated using the AMBER22 LEaP module, and the residue-specific ff19SB force field was employed. As a result, coordinate and topology files were built for the parameters of protein residues^42^. An OPC (optimal point charge) water model with a 12 Å buffer was used to solvate each system in a truncated octahedral box. A 1 Å grid of monovalent OPC ions (Na^+^ and Cl^−^) with a concentration of 0.1 M was utilized to determine the system's neutrality^43^. The complexes of α-glucosidase small inhibitors structure were adjusted using the General Amber Force Field-2 (GAFF2). Small-molecule AM1-BCC charges were calculated to refine the geometry^44^. Hydrogen atoms that were missing were added using the LEaP module, and AMBER22's Parmchk2 tool was used to produce the necessary force field parameters for the small compounds in the complexes^45^. Hydrogen atom locations were constrained using the SHAKE algorithm^46^ during the MD simulation, allowing a time step of 2 fs. Long-range electrostatic interactions were calculated using the particle-mesh Ewald method (PMEMD)^8^ with a threshold of 8 Å. GPUs were used to simulate maximum parallel scalability. The complex systems underwent two minimization cycles following MD initialization to refine the structure. In the first iteration of optimization, constraints were placed on the protein residues, and the system was subjected to 50,000 steps of steepest descent minimization. The conjugate gradient algorithm was then in the second minimization with a step size of 25,000^47^. The systems were heated gradually using a Langevin thermostat^46^. Weak restraints were utilized to keep the protein in place as the system was heated from 0.1 to 300 K throughout 400 picoseconds. The heating relied on an NVT ensemble, where N is the number of particles, V is the volume of the simulation box, and T is the temperature. The harmonic oscillator's kinetic energy was regulated by the 2.0 ps^−1^ collision frequency with the Langevin thermostat^48^. The system's density was then modified the same way as the heating phase in a 400 ps time scale. After the system reached 300 K, a 2000 ps equilibration operation was performed in a constraint-free NPT ensemble (where N is the number of particles, P is the pressure, and T is the temperature). An isotropic position scaling approach was employed to maintain a consistent pressure during the equilibration phase. This made it possible to attain a relaxation time of 2 picoseconds. The free state, the benchmark complex (alpha-D-glucopyranose-glucosidase complex), and the best three docked protein-inhibitor complexes were all subjected to a 100 ns production MD simulation in an NPT ensemble. The temperature was maintained by a Langevin thermostat, and the electrostatic interactions were computed using a cutoff distance of 8 Å. Each system's output trajectory was collected after10 ps simulation time step.

### Evaluation of post-dynamic trajectory

#### Investigation of protein stability

The CPPTRAJ module in AMBER22 was used to evaluate the 100 ns trajectory data produced by the simulation of each system^49^. Root Mean Square Deviation (RMSD) profiles were generated for each system based on the Cα atoms of the protein, using data from 11,000 frames of trajectories. The RMSD was computed using the first trajectory frame as the coordinate reference. The average RMSD was calculated for each trajectory to examine how the RMSD changed over time.

#### Investigating residue fluctuations and compactness

When calculating the distances that each amino acid position shifted from its initial position, the Root Mean Square Fluctuation (RMSF) was used^49^. Protein residue flexibility is shown by the RMSF values calculated. During a 100 ns simulation, the RG of the protein was determined to determine how compact the protein was before and after ligand binding. RG uses the equation from^50^ to determine the positions of the protein's atoms concerning its mass centroid. We calculated the amount of pucker in the five-membered ring using the method developed by Altona and Sundaralingam^13^. In addition, cyclic averages in Rg calculations are depicted for periodic torsions.

#### Binding free energy calculations

Computational methods have been developed to determine the binding free energy (BFE) of protein–ligand complexes in recent years. There has been much research on the efficacy and applicability of these techniques^51–53^ to different protein–ligand systems. Free energy is broken down into constituent energy contributors that result from different interactions, providing remarkable accuracy at a computationally affordable price. THE BFE between protein and ligand complexes was calculated using AMBER22's Molecular Mechanics/Generalized Born Surface Area (MM/GBSA) method^14,17,18^. The BFE was computed using the latest 2750 frames (25 ns) of the roduction trajectry for each complex. The idea topology of the systems was generated using the mbondi3 radii and a solvent probe with a 2 Å radius. We calculated the protein–ligand complexe' BFE (ΔG_bind_) using the following equation^54^

3 $$\begin{array}{c}\Delta {G}_{bind}= {\Delta G}_{R+L}-\left(\Delta {G}_{R}+{\Delta G}_{L}\right)\end{array}$$

To calculate the binding free energy (BFE), first, we subtract the free energies of the free ligand (ΔG_L_) and the free protein (ΔG_R_) from the free energy of the protein–ligand complex (ΔG_R+L_). The free energy component (G) in the equation may be decomposed into many energetic contributions using the Molecular Mechanics/Gauss-Boltzmann Surface Area (MM/GBSA) and Molecular Mechanics/Poison Boltzmann Surface Area (MM/PBSA) methods (Eq. 4).

4 $$\begin{array}{c}G={E}_{bond}+{E}_{VDW}+{E}_{elec}+{G}_{GB}+{G}_{SA}-T{S}_{S}\end{array}$$

Overall bond energy, or E_bond_, is the sum of a molecule's bond energy and the angle and dihedral energies. Evdw represents Van der Waals's contribution, whereas electrostatic energy is represented by the sign E_elec_. Polar (G_GB_) and non-polar (G_SA_) components comprise the solvation energy. The non-polar component is estimated in SASA through the LCPO method, which represents a linear pairwise overlap combination. However, the GB model determines the polar component^55^. The energy was calculated in kcal/mol, whereas Å^2^ was used to estimate the protein's surface area. Absolute temperature T and solute entropy S_S_ were calculated. We calculated the free energy of four distinct complex systems using the MM/PB(GB)SA model. The α-glucosidase-alpha-D-glucopyranose complex (3A4A) and three α-glucosidase-inhibitor complexes were among these systems.

#### Data examination

The structural drawings were constructed using the software programs MOE2022.02^21^ and Blender^22^. OriginPro, a graphing application, was used^56^.

## Conclusion

Several multifactorial diseases are associated with diabetes mellitus and its complications, necessitating advancements in drug discovery for the prevention and treatment of diabetes-based issues. In the present study, a series of coumarin-based drug-like molecules were designed as drug candidates for diabetes mellitus. Their antidiabetic potential was assessed by evaluating their ability to block the α-glucosidase enzyme function, and all the compounds displayed excellent α-glucosidase inhibitory potential with IC_50_ values ranging from 2.33 to 22.11 µM. The concentration-dependent inhibition of compound **3c, 3g** and **3i** was studied by Kinetic studies. Furthermore, the binding pattern of these molecules at the atomic level was examined through molecular docking, which revealed that thiosemicarbazide moiety plays a crucial role in binding these molecules to the catalytic and active site residues of the enzyme. However, the conformational changes in these molecules are primarily responsible for their diverse bioactivity. The molecular dynamic simulations explored the firm attachment of the compounds to the protein’s active site and showed an impact on the protein structural confirmation.

### Supplementary Information

Supplementary Information.

## Data Availability

All data generated or analyzed during this study are included in this published article [and its supplementary information files].
