# Supplementary material for: Synthesis of novel coumarin-based thiosemicarbazones and their implications in diabetic management via in-vitro and in-silico approaches
Source: Sci Rep. 2023 Oct 21;13:18014. doi: 10.1038/s41598-023-44837-6 (PMC10590377; doi:10.1038/s41598-023-44837-6)
Supplement: Supplementary file 1 — Supplementary Information. [file 41598_2023_44837_MOESM1_ESM.docx]

***Supplementary Material***

**Synthesis of novel coumarin-based thiosemicarbazones and their implications in diabetic management via *in-vitro* and *in-silico* approaches**

Syeda Bakhtawar Zahra^a^, Saeed Ullah^b^, Sobia Ahsan Halim^b^, Noor Ul Huda^a^, Muhammad Waqas^b^ Ajmal Khan^b^, Ammena Y. Binsaleh^c^ , Attalla F. El-kottd^d,e^, Javid Hussain^f^*, Ahmed Al-Harrasi^b^* and Zahid Shafiq^a^*

*^a^Institute of Chemical Sciences, Bahauddin Zakariya University, Multan-60800, Pakistan*

*^b^Natural and Medical Sciences Research Centre, University of Nizwa, P.O. Box 33, PC 616, Birkat Al Mauz, Nizwa, Sultanate of Oman*

*^c^Department of Pharmacy Practice, College of Pharmacy, Princess Nourah bint Abdulrahman University, P.O. Box 84428, Riyadh 11671, Saudi Arabia*

*^d^Department of Biology, College of Science, King Khalid University, Abha 61421, Saudi Arabia*

*^e^Department of Zoology, College of Science, Damanhour University, Damanhour 22511, Egypt*

*^f^Department of Biological Sciences & Chemistry, College of Arts and Sciences, University of Nizwa 616, Oman*

**Corresponding Author*:

Prof. Dr. Zahid Shafiq: [zahidshafiq@bzu.edu.pk](mailto:zahidshafiq@bzu.edu.pk)

Prof. Dr. Ahmed Al-Harrasi: aharrasi@unizwa.edu.om

**H^1^ & ^13^CNMR of 3a**

**
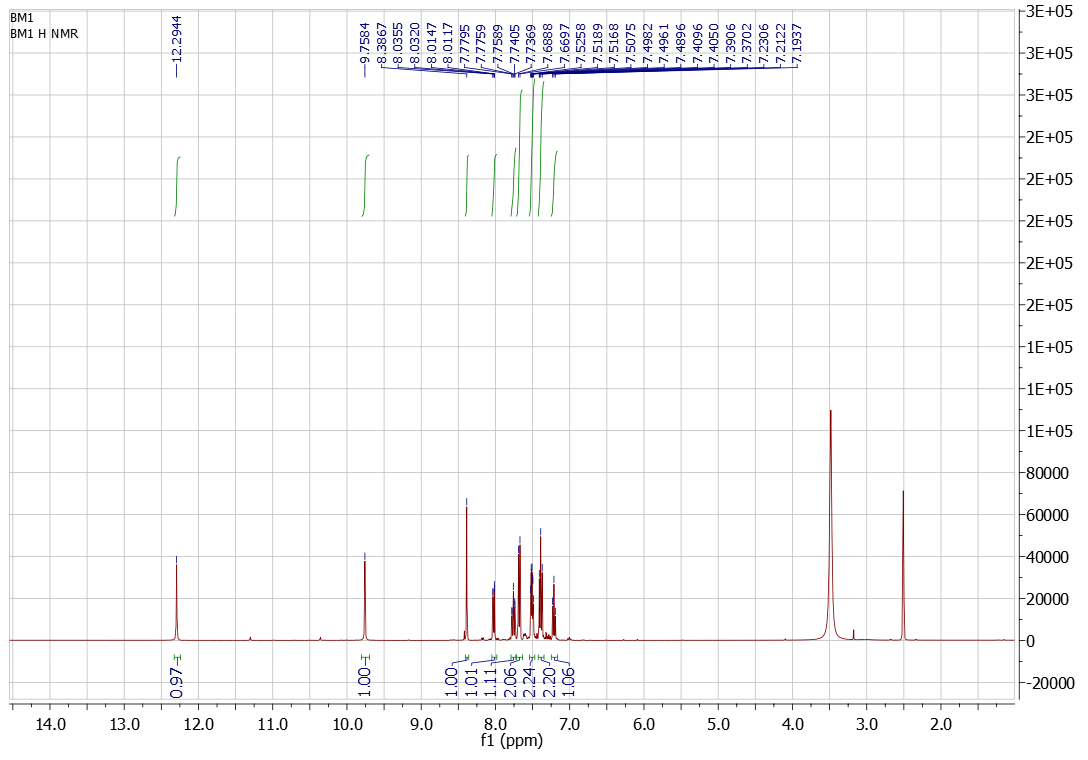

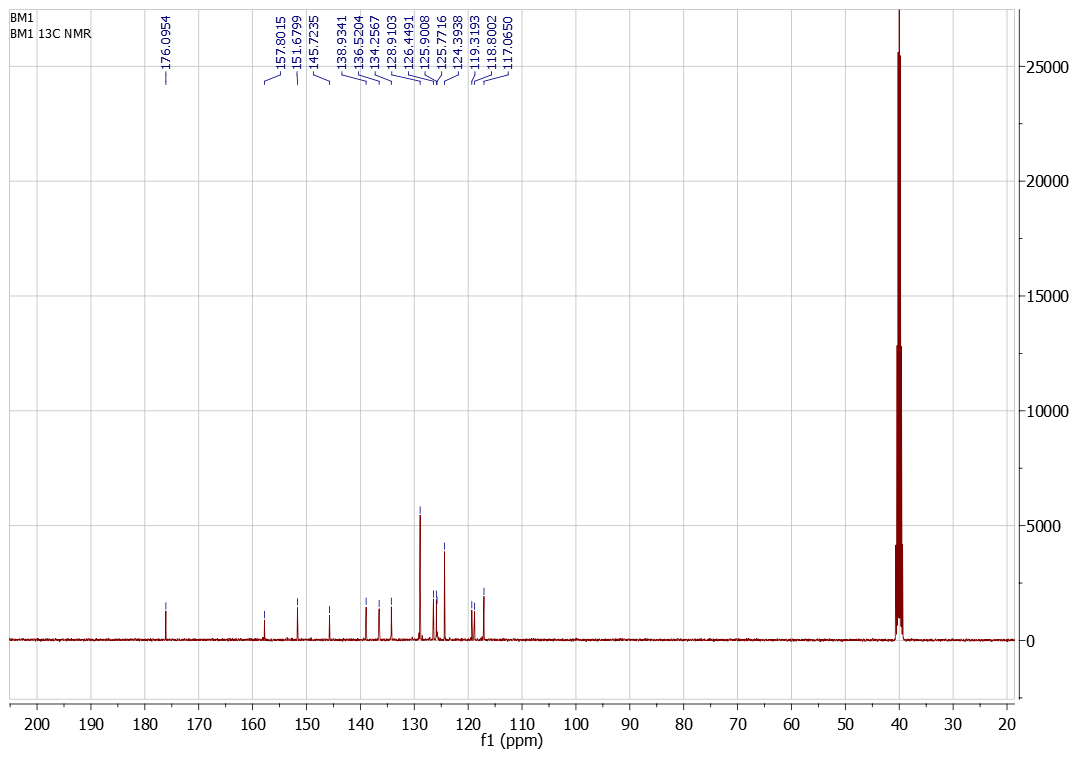
**

**H^1^ & ^13^CNMR of 3b**

**
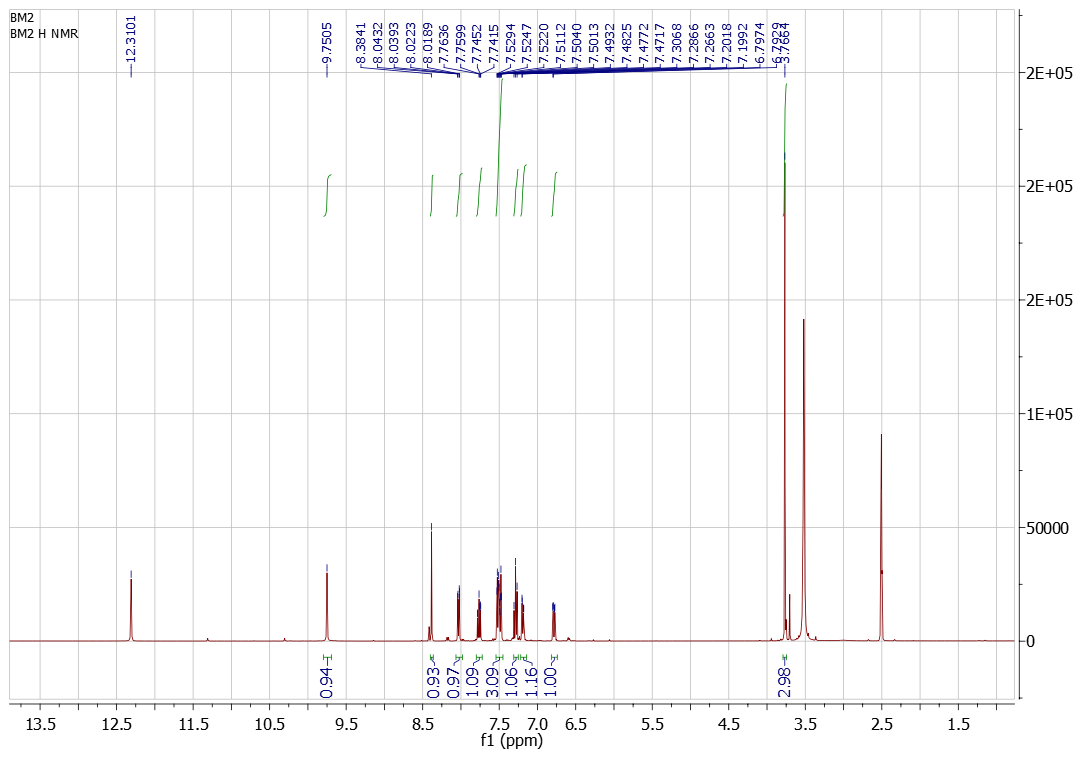
** **
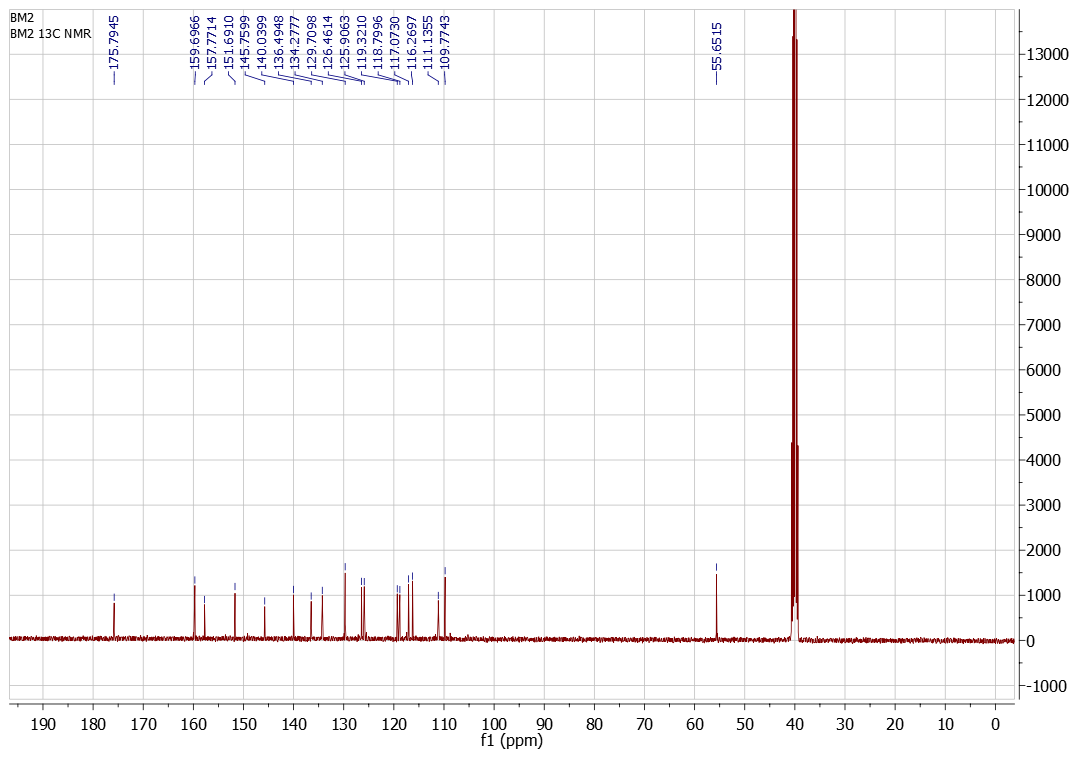
**

**H^1^ & ^13^CNMR of 3c**

**
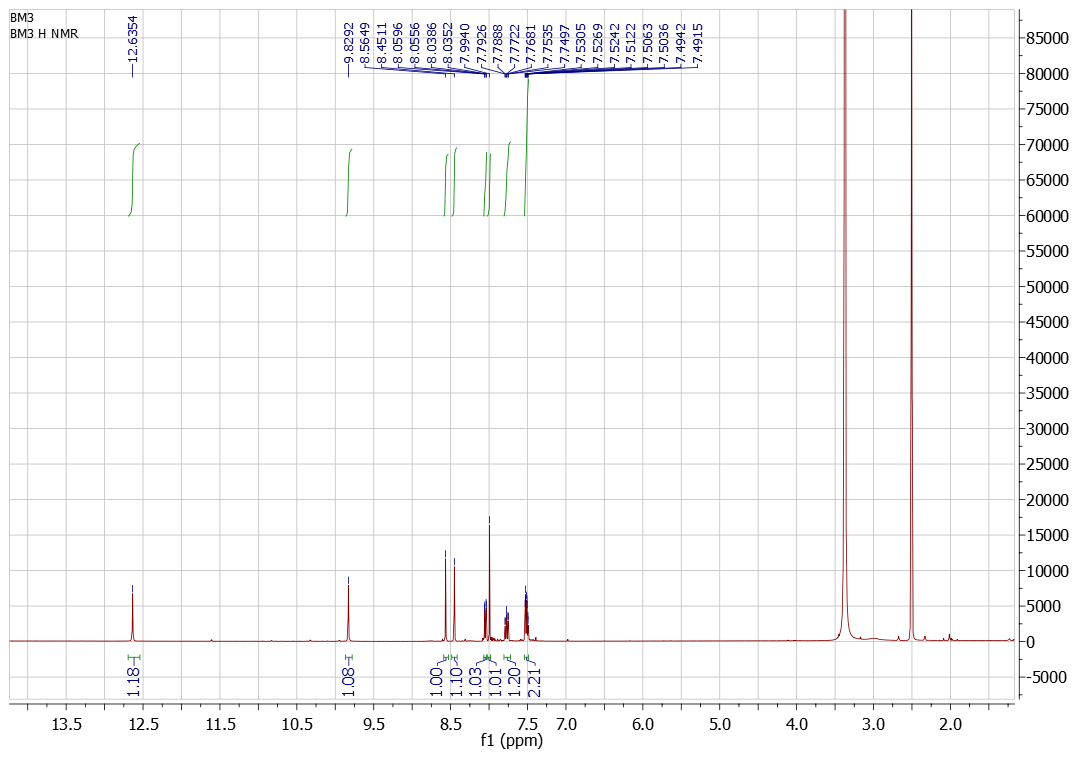
** **
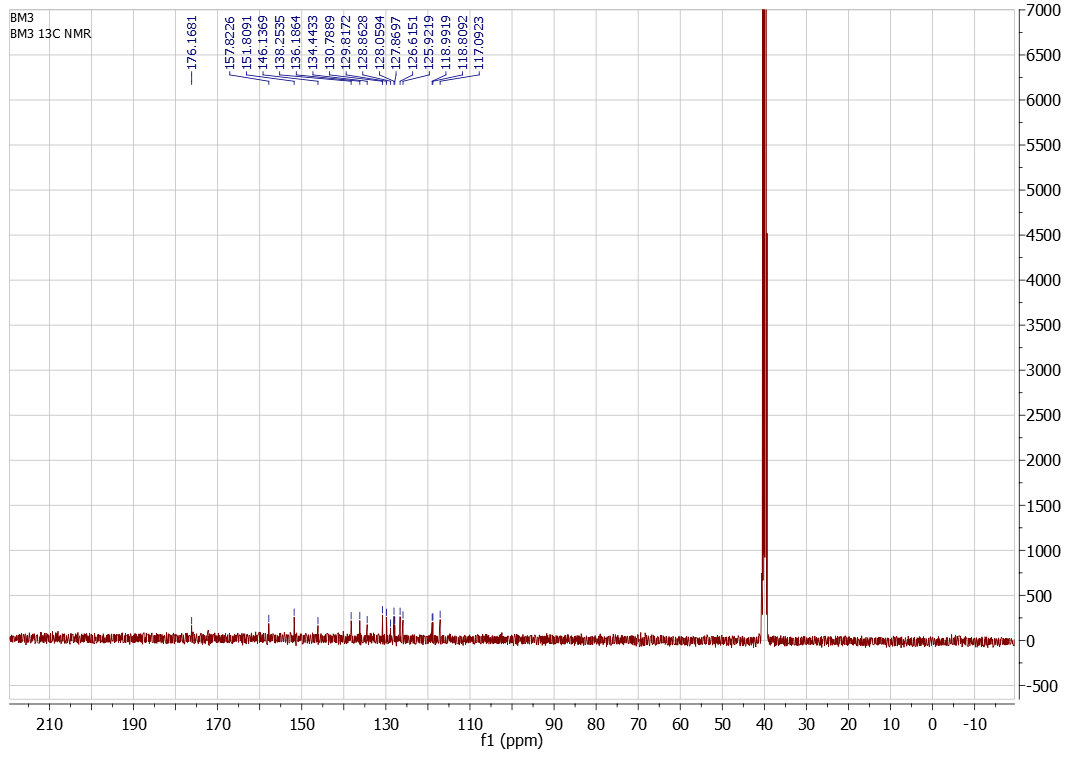
**

**H^1^ & ^13^CNMR of 3d**

**
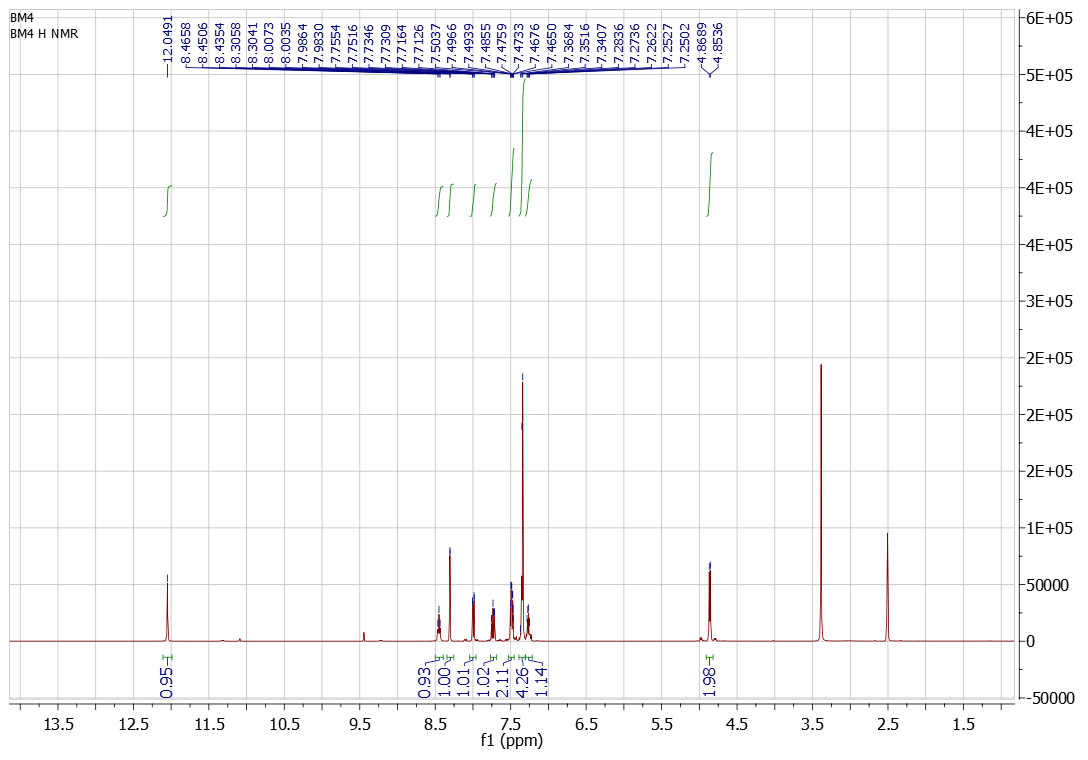
** **
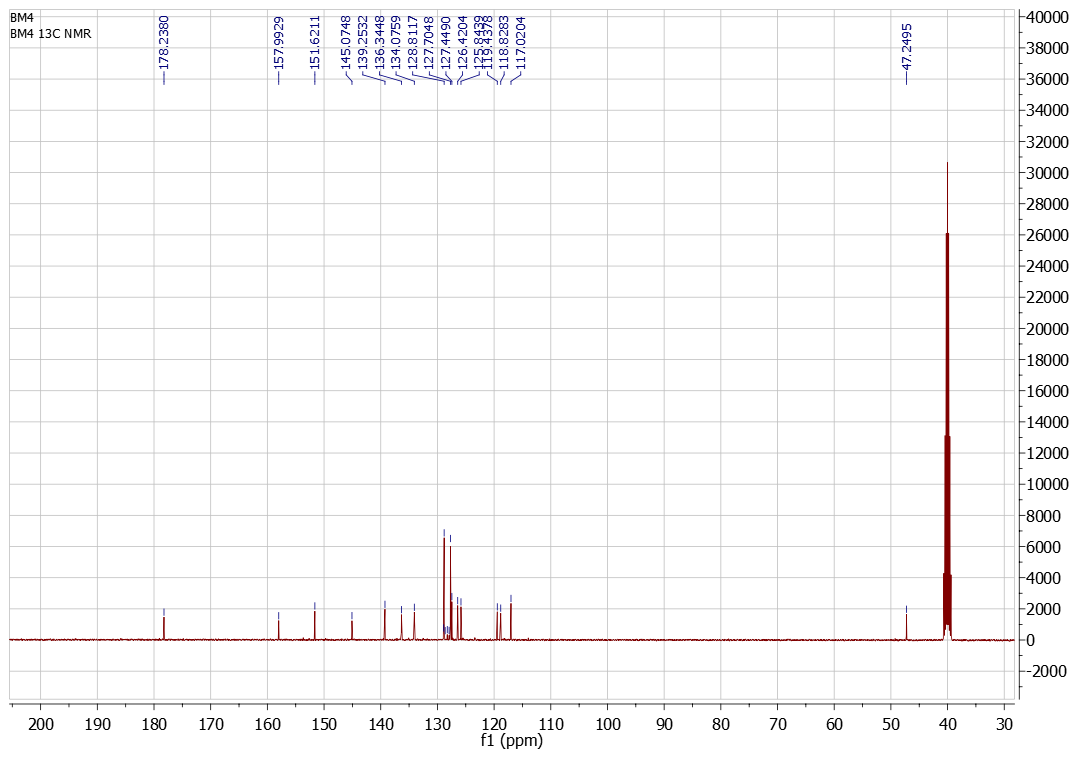
**

**H^1^ & ^13^CNMR of 3e**

**
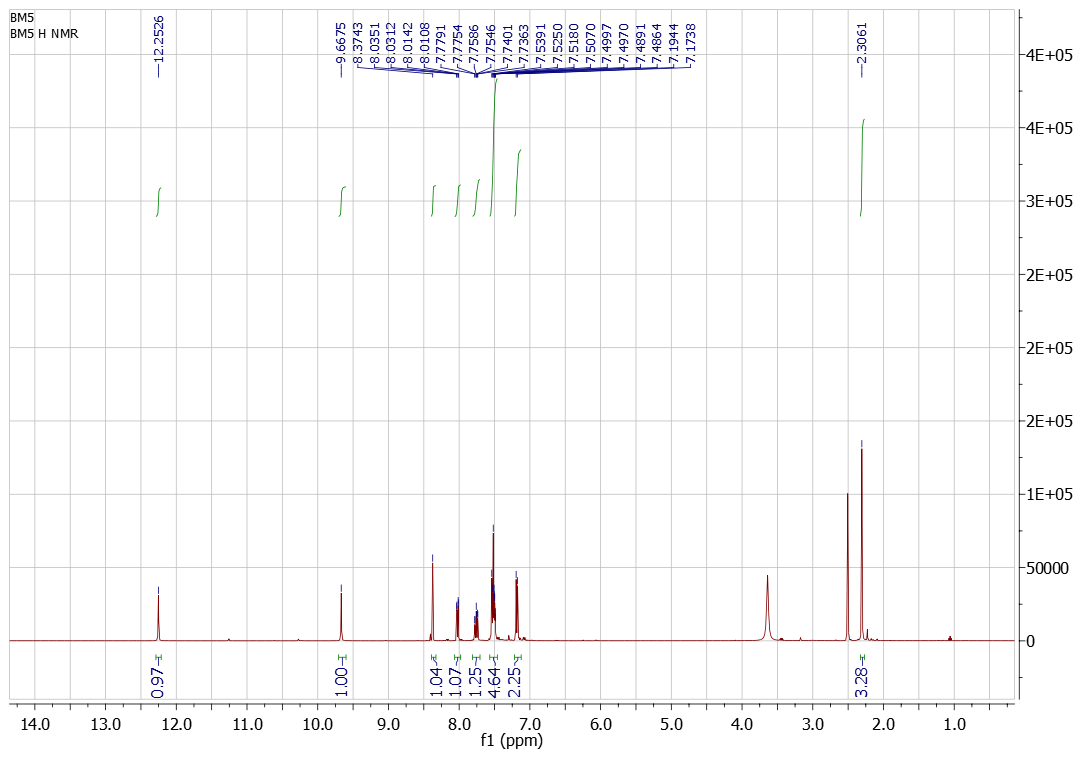
** **
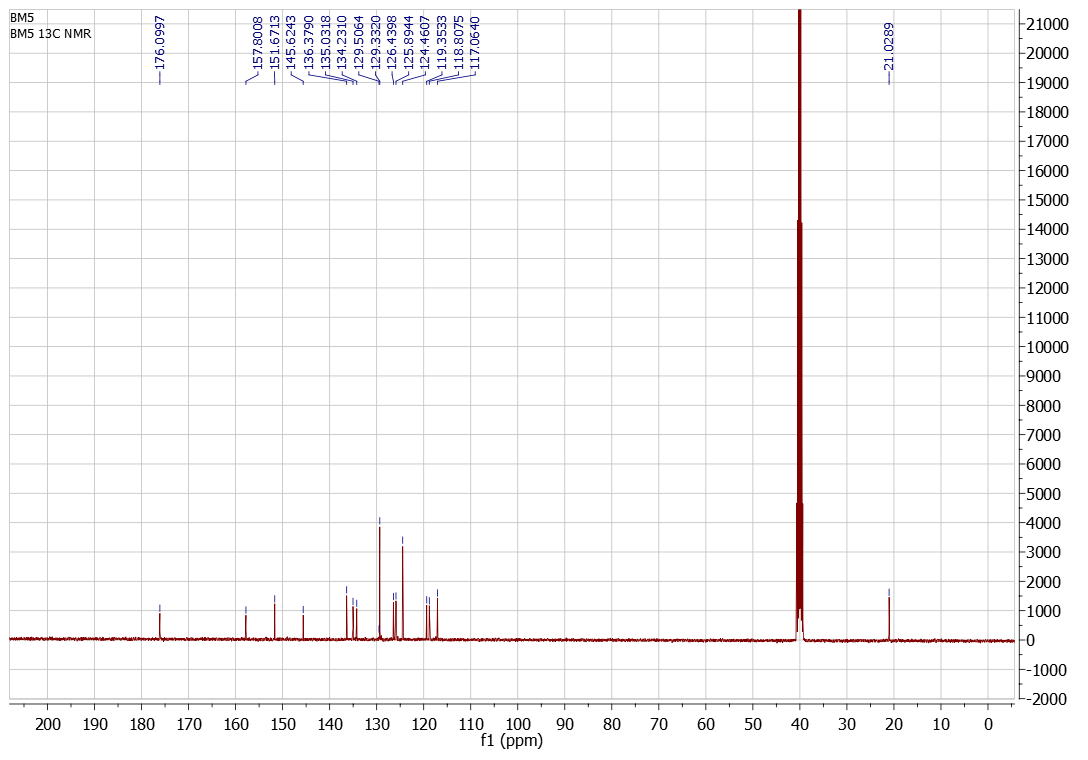
**

**H^1^ & ^13^CNMR of 3f**

**
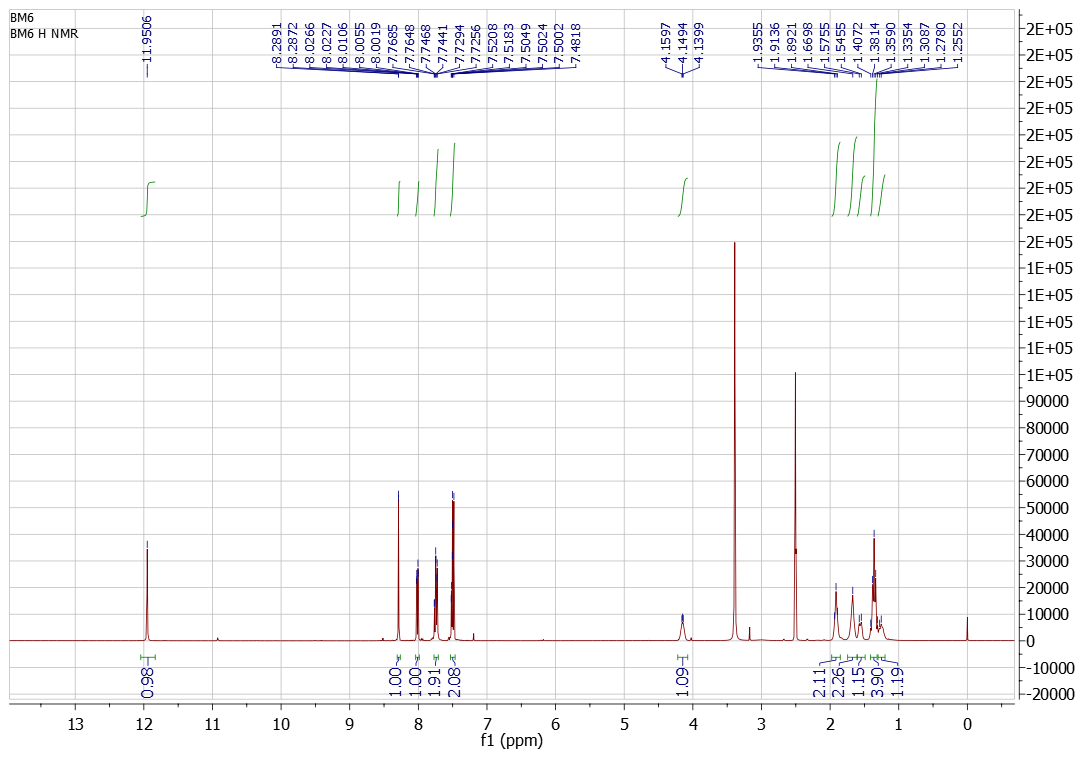
** **
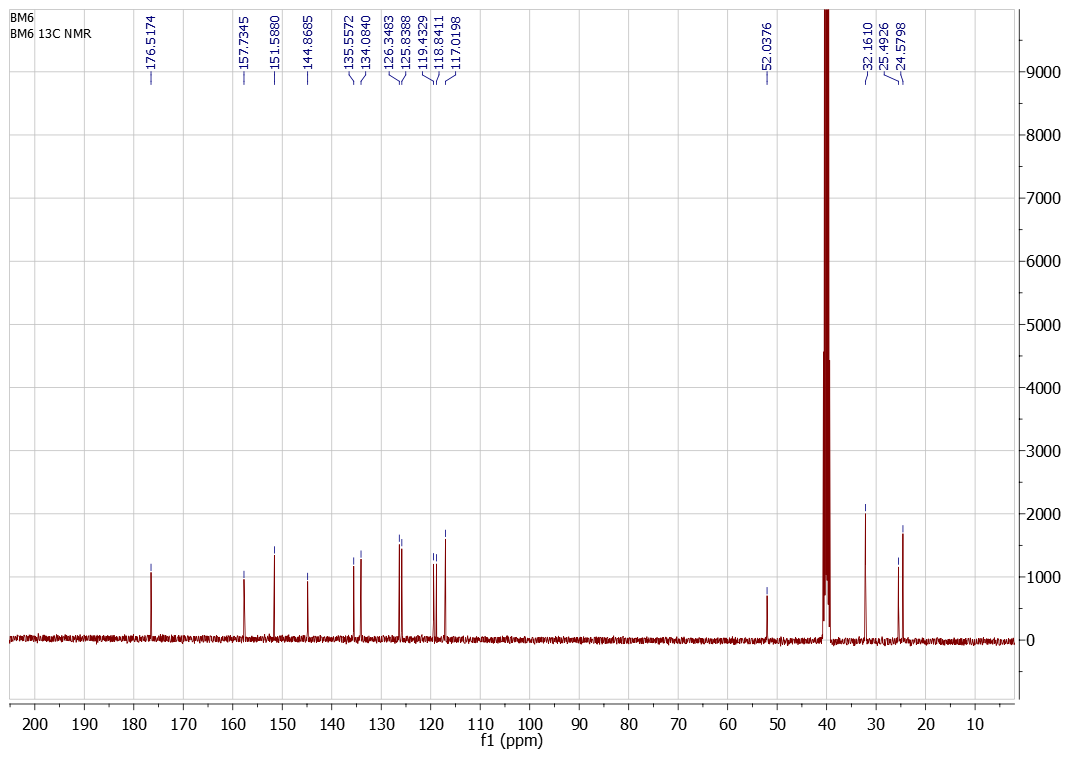
**

**H^1^ & ^13^CNMR of 3g**

**
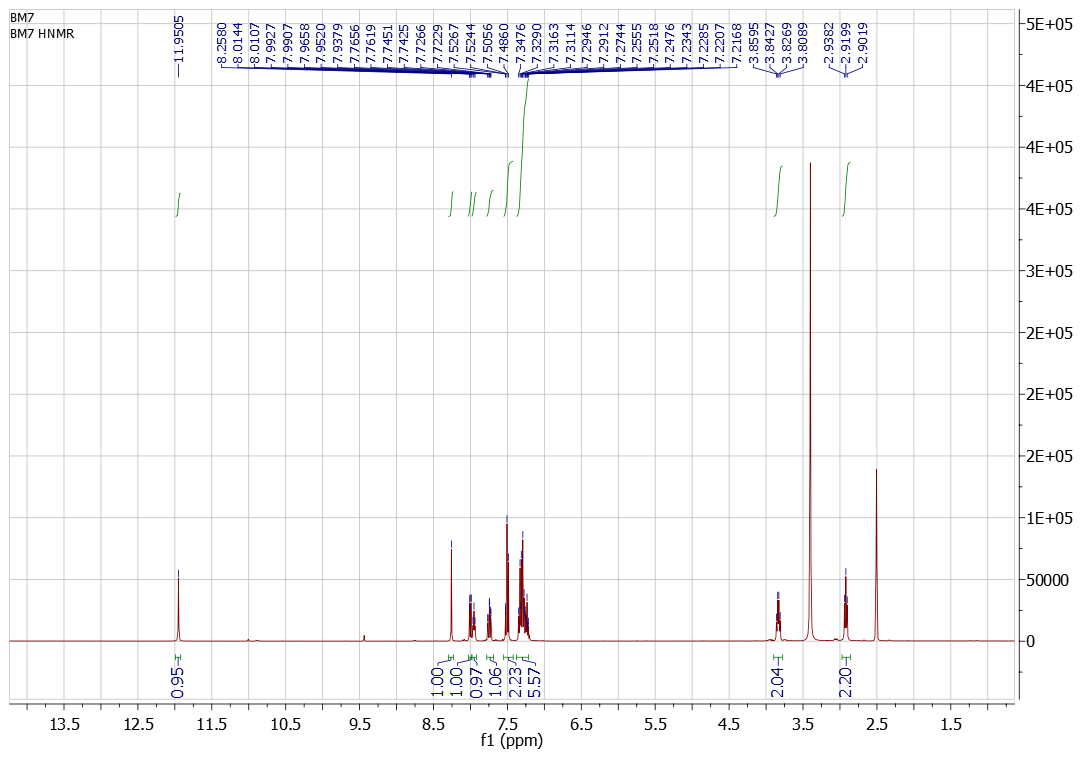
** **
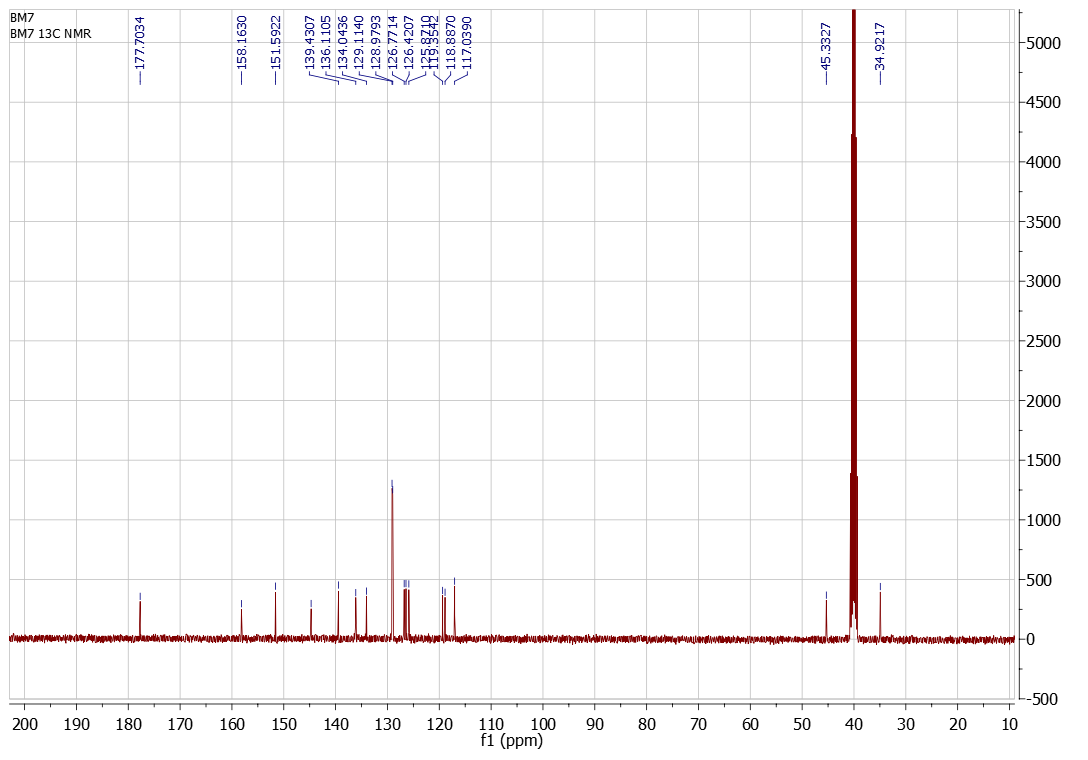
**

**H^1^ & ^13^CNMR of 3h**


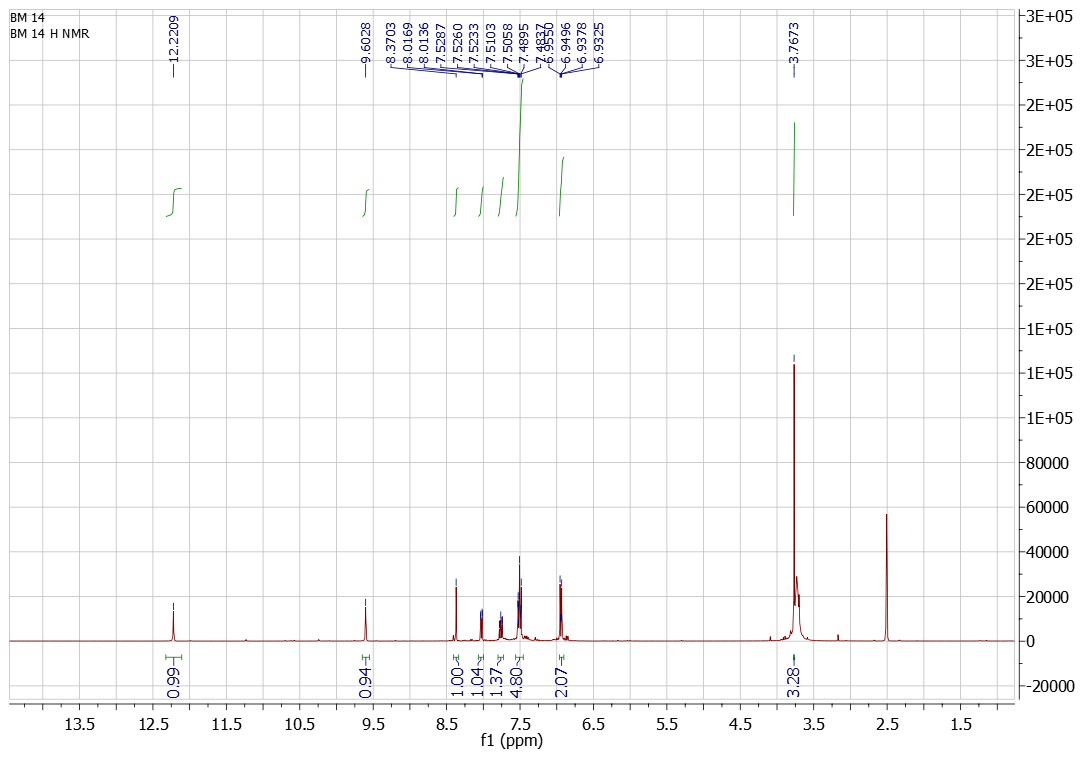

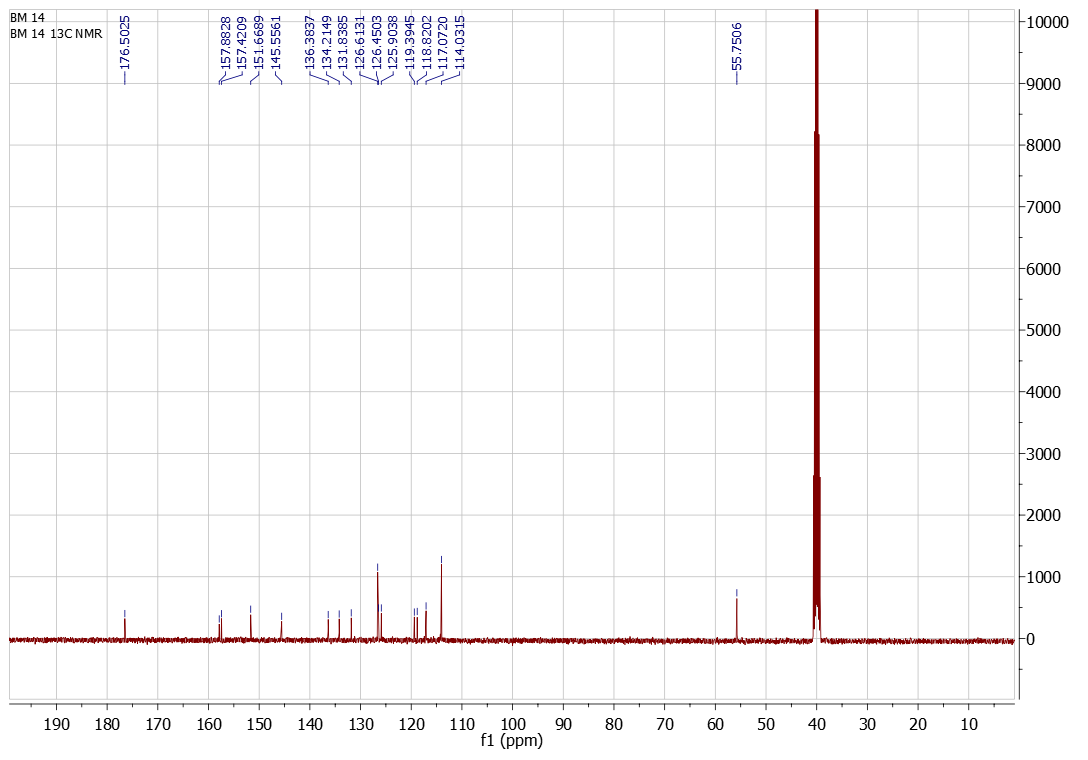


**H^1^ & ^13^CNMR of 3i**


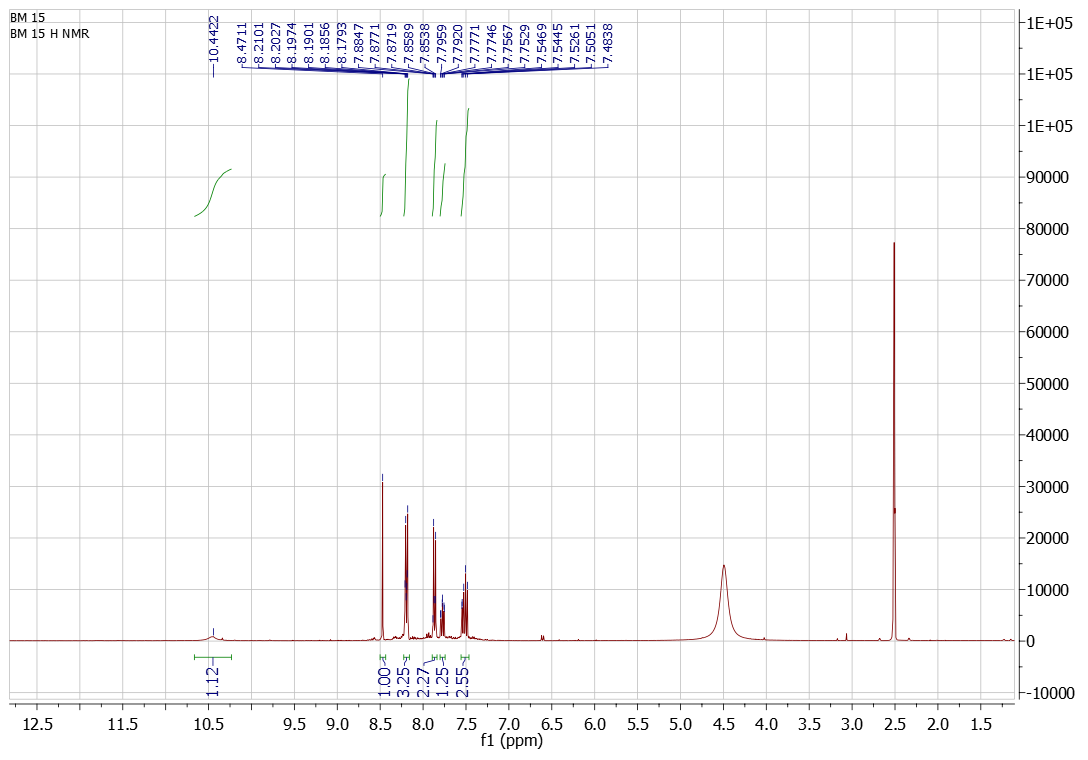

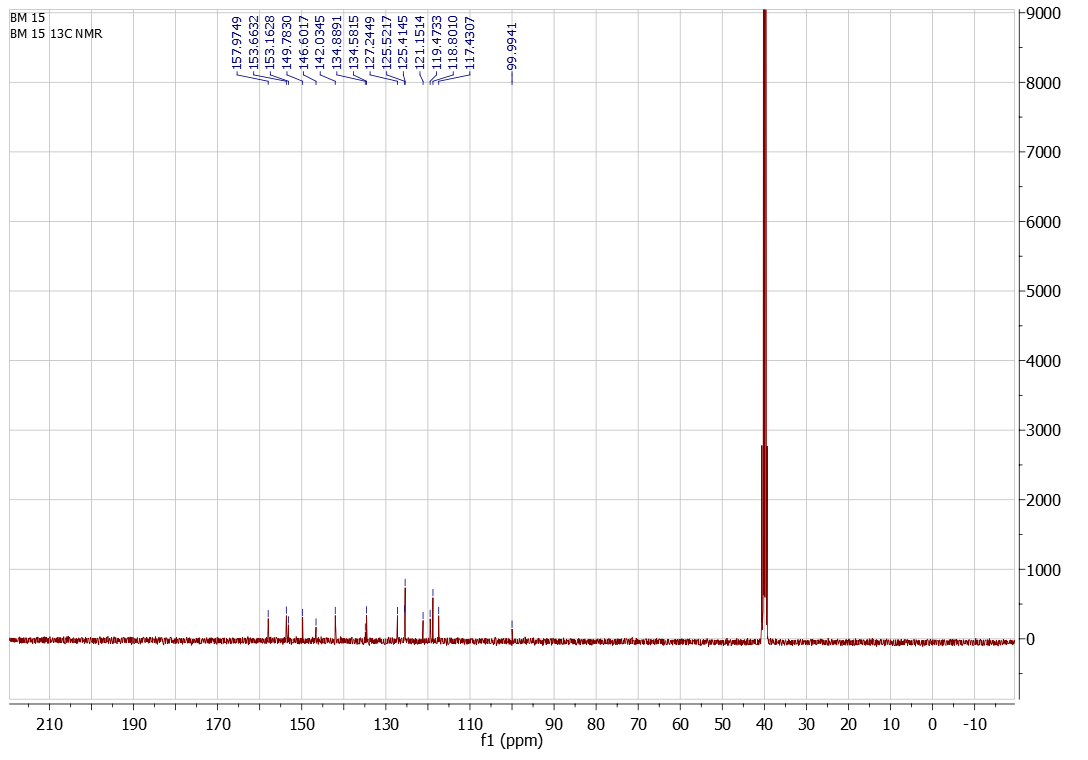


**H^1^ & ^13^CNMR of 3j**

**
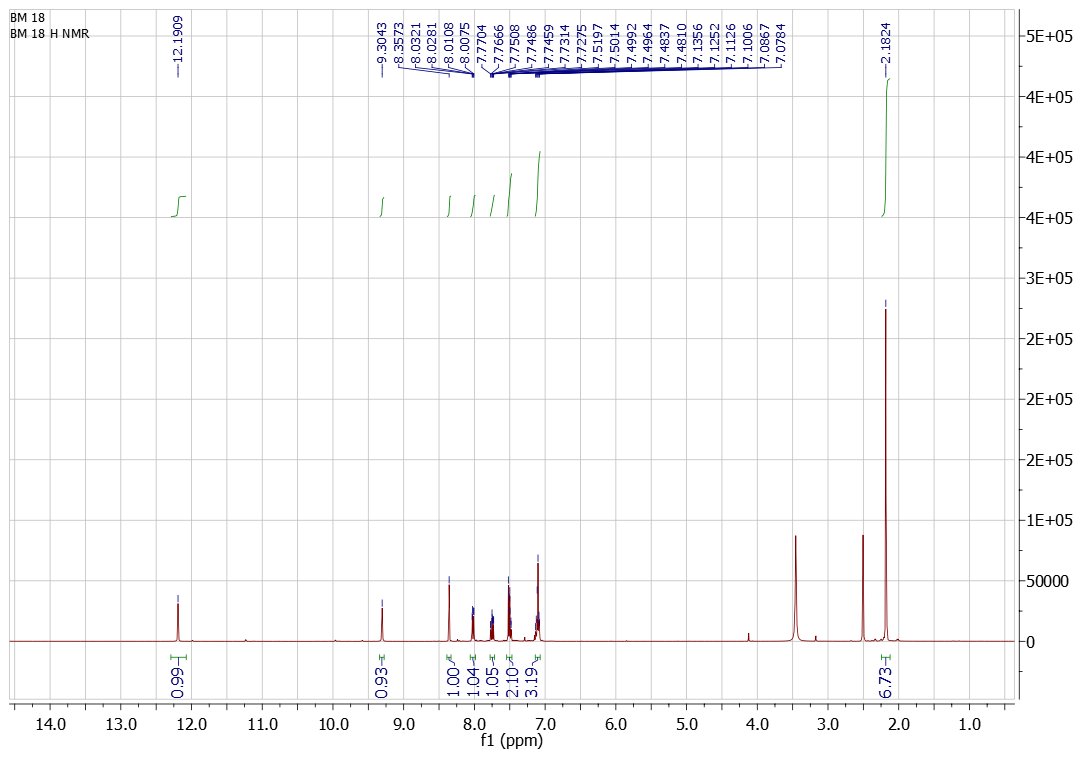
** **
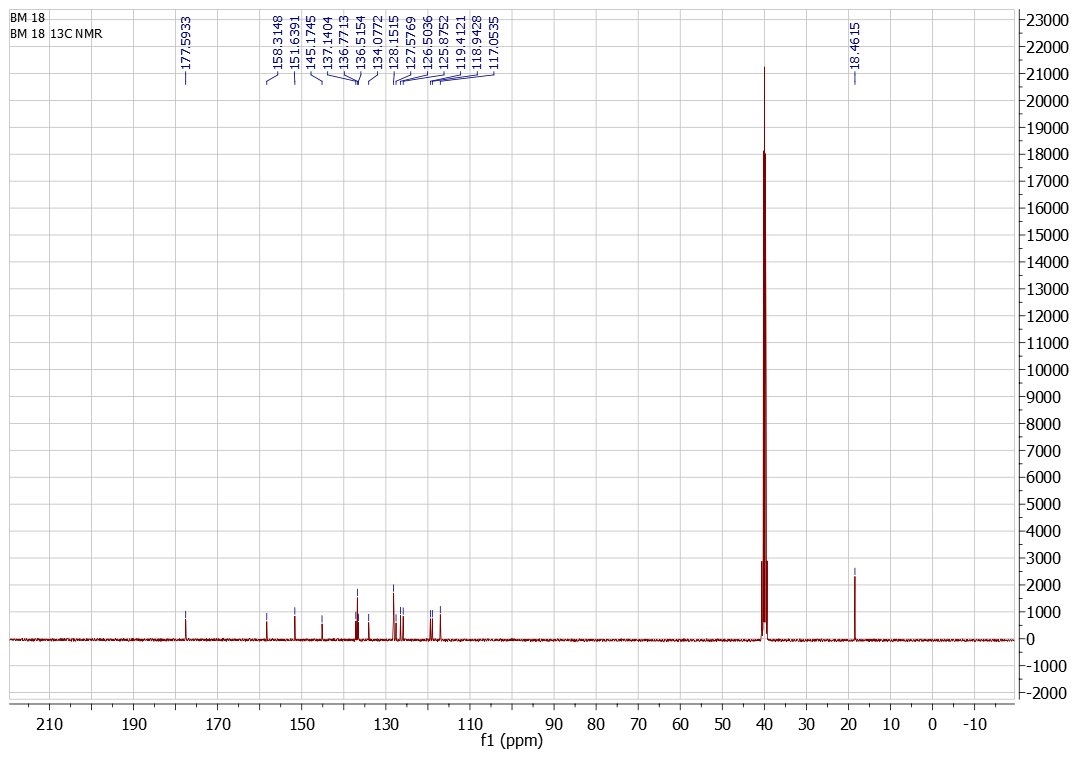
**

**H^1^ & ^13^CNMR of 3k**

**
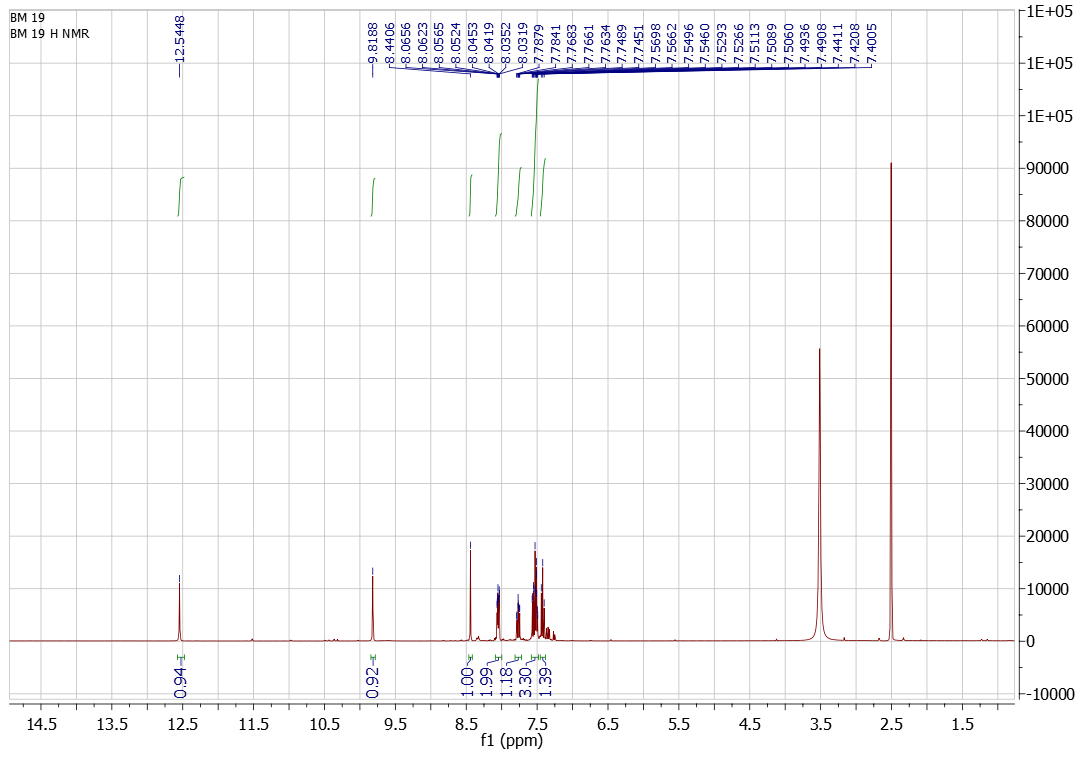
** **
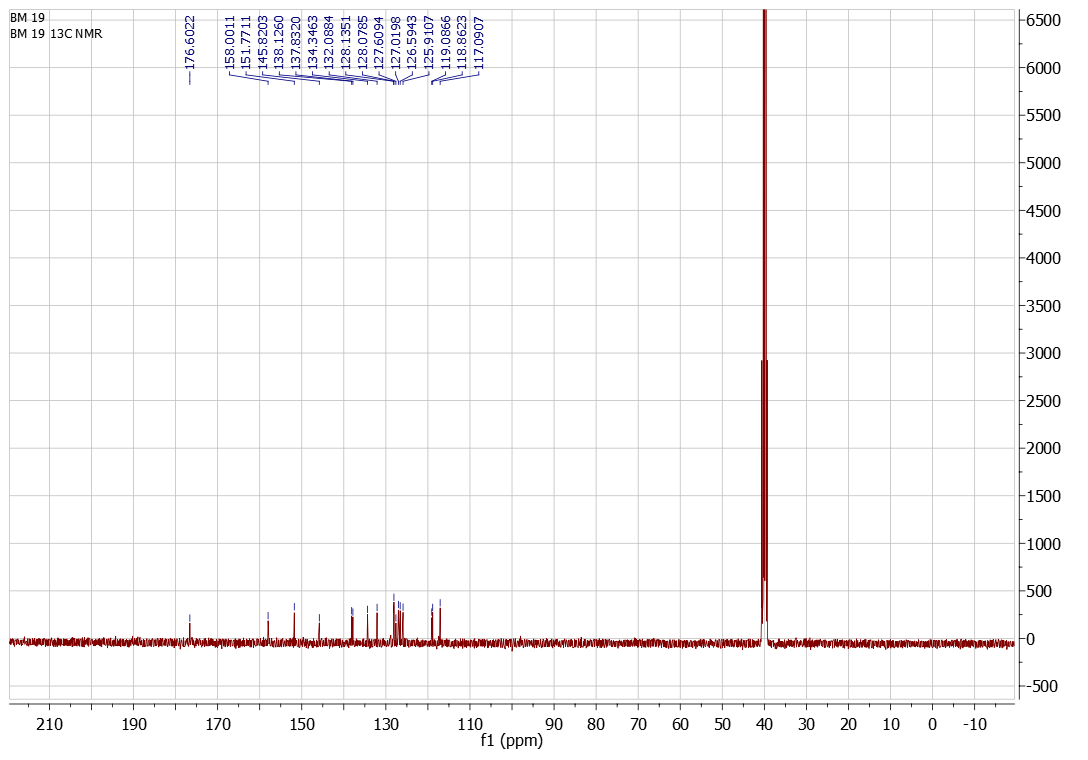
**

**H^1^ & ^13^CNMR of 3l**

**
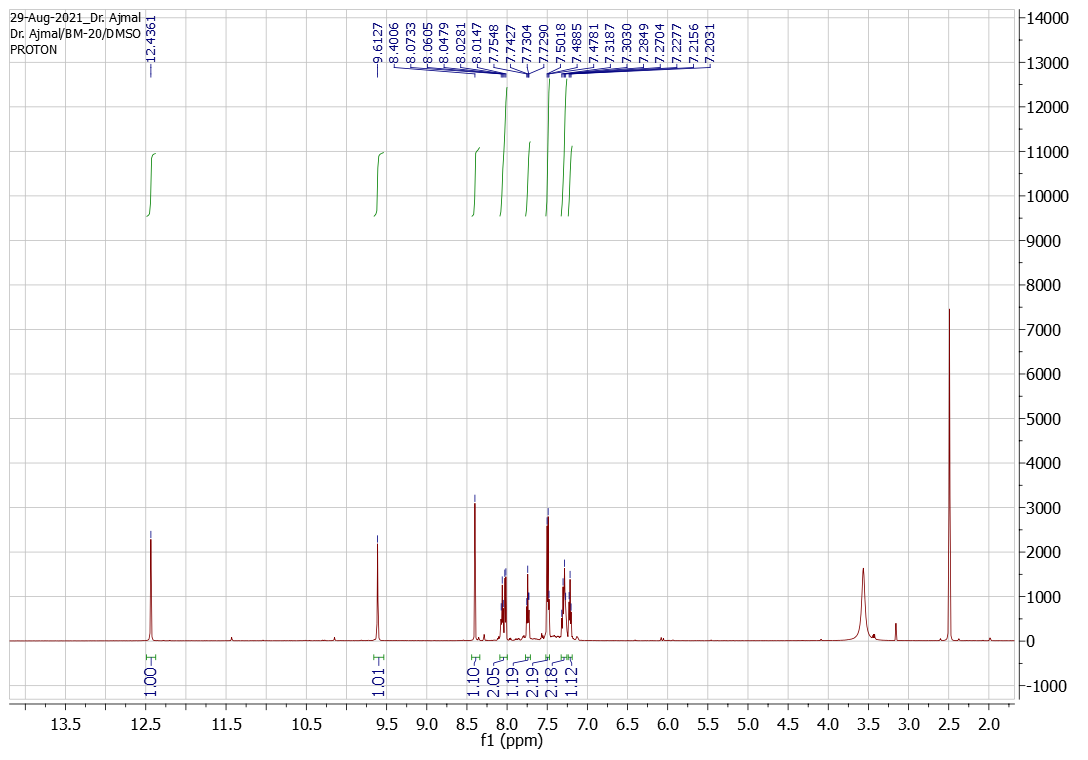
** **
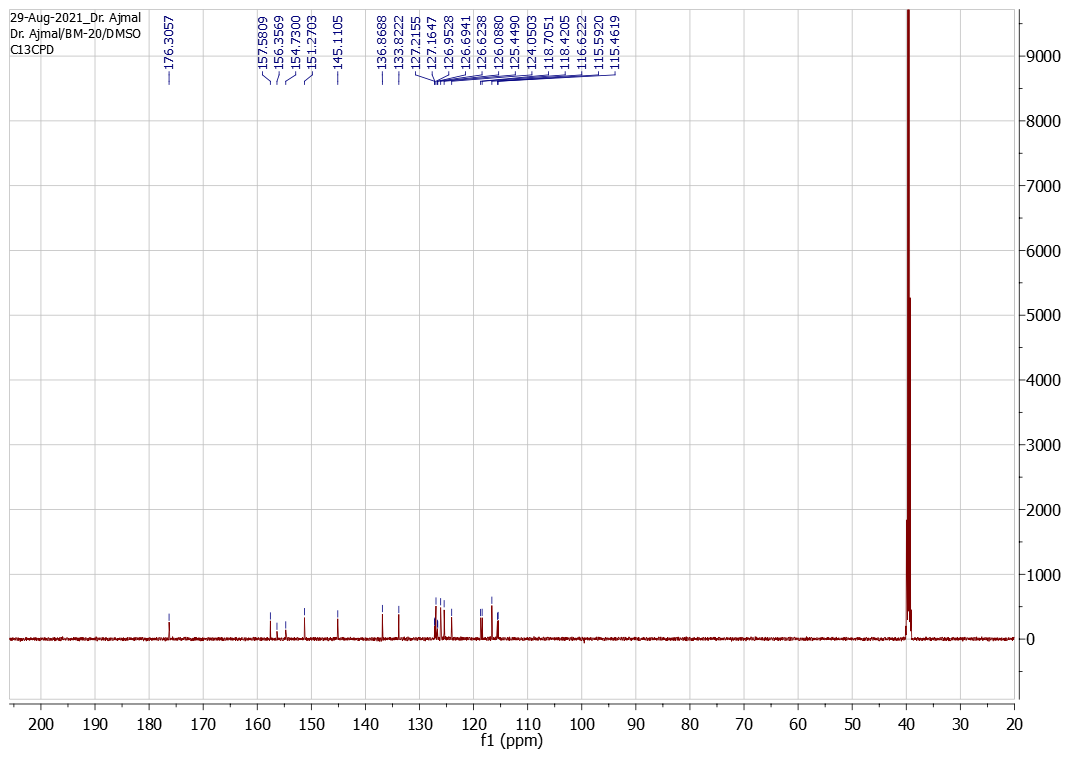
**

**H^1^ & ^13^CNMR of 3m**

**
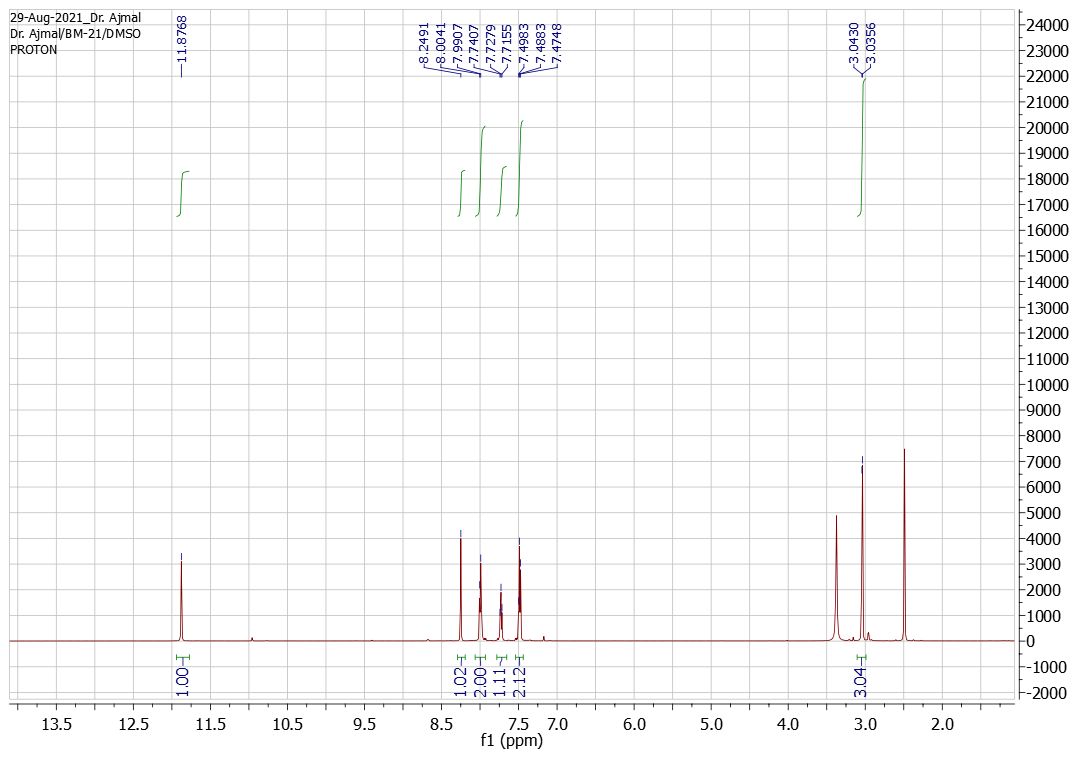
** **
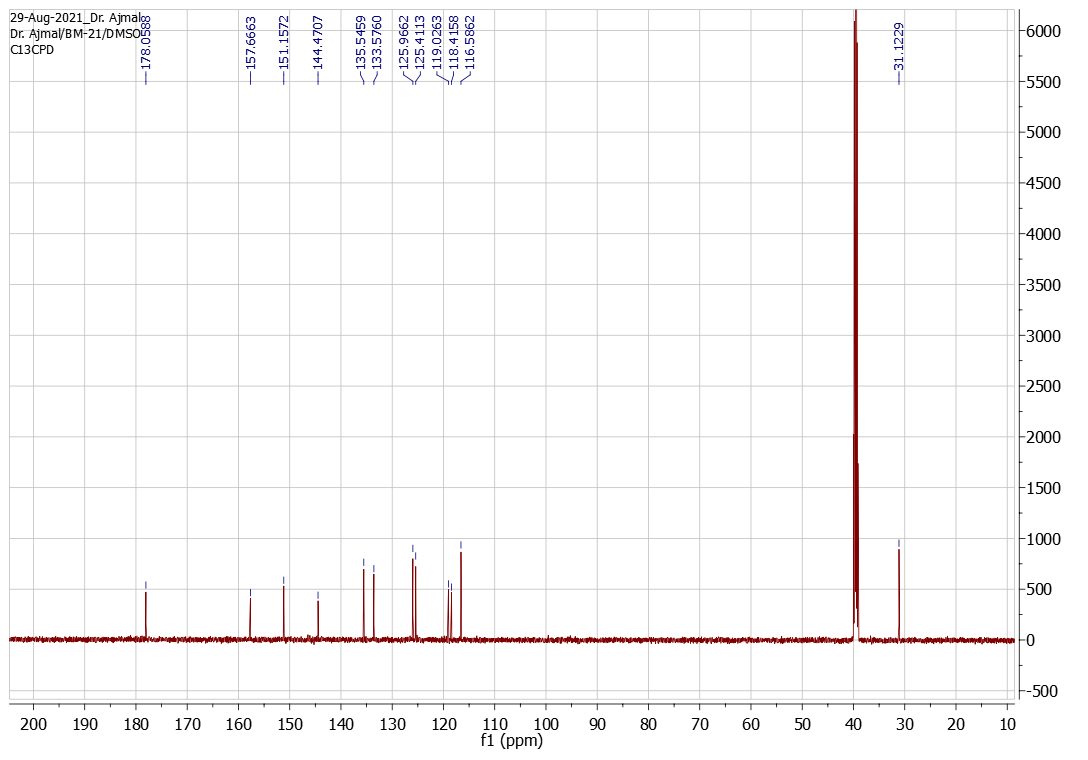
**

**
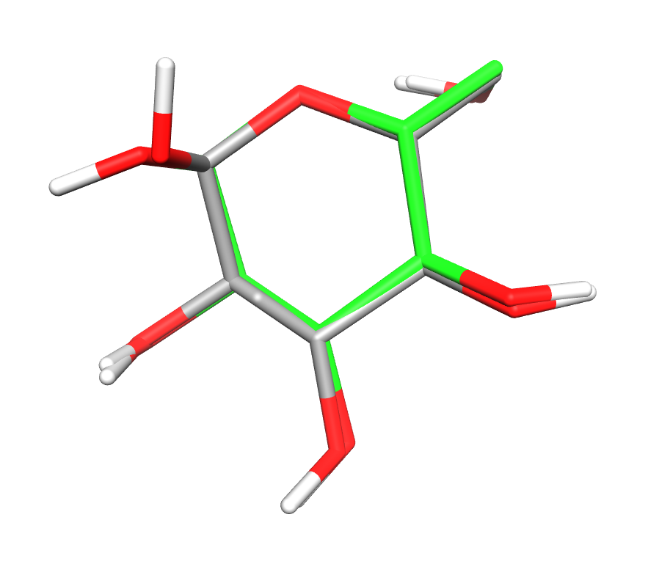
**

**Figure S1. The superimposed view of X-ray crystal structure of isomaltase (grey sticks) and its re-docked conformation (shown in green sticks) with RMSD value of 0.16Å.**
